# The effects of exercise intervention on children with developmental coordination disorder: a systematic review and network meta-analysis of randomized controlled trials

**DOI:** 10.3389/fphys.2026.1877672

**Published:** 2026-07-07

**Authors:** Yanfeng Dong, Shuan Xue, Jiping Chen, Rumeng Liu, Xiangli Xue

**Affiliations:** 1 School of Physical Education, Shandong University, Jinan, China; 2 College of Health Sciences, Shandong University of Traditional Chinese Medicine, Jinan, China

**Keywords:** child, developmental coordination disorder, exercise, motor skills, network meta-analysis, postural balance

## Abstract

**Background:**

Motor interventions are widely used to improve motor function in children with developmental coordination disorder (DCD), but their relative efficacy across key functional domains remains unclear. This network meta-analysis aimed to compare the effects of integrated training (IT), task-oriented training (TOT), and process-oriented training (POT).

**Methods:**

Randomized controlled trials (RCTs) published between January 2000 and January 2026 were systematically retrieved from multiple databases following PRISMA 2020 and PRISMA−NMA reporting standards. Multiple validated motor assessment tools were harmonized for pooled analysis. Standardized mean differences (SMDs) with 95% confidence intervals (CIs) were calculated, and interventions were ranked using the surface under the cumulative ranking curve (SUCRA). Subgroup and sensitivity analyses were performed.

**Results:**

Twenty RCTs (n = 1,867) were included. All three interventions were associated with significant improvements in motor function outcomes. TOT showed the largest estimated effect on overall motor function (SMD = 2. 30, 95% CI: 0. 42 to 4. 18) and achieved the highest SUCRA ranking (55. 3%). IT achieved the highest SUCRA rankings for balance (SMD = −1. 93, 95% CI: −3. 10 to −0. 76; SUCRA = 78. 3%) and manual dexterity (SMD = −2. 11, 95% CI: −4. 12 to −0. 10; SUCRA = 71. 5%). For aiming and catching, IT (SMD = −3. 90, 95% CI: −7. 51 to −0. 29) and POT (SMD = −3. 83, 95% CI: −7. 48 to −0. 18) showed significant improvements, whereas TOT did not demonstrate a significant effect. Interventions lasting more than 8 weeks were associated with greater effects (P = 0. 001), while age was not a significant moderator (P = 0. 577). Sensitivity analyses supported the robustness of the overall findings, although significant small-study effects were observed for several outcomes; incomplete blinding and substantial cross-study heterogeneity may inflate pooled effect sizes.

**Conclusion:**

The available evidence suggests that motor interventions may improve motor function in children with DCD, with potentially different benefits across functional domains. TOT achieved the highest ranking probability for overall motor function, whereas IT achieved the highest rankings for balance and manual dexterity. In addition, both IT and POT were associated with significant improvements in aiming and catching ability. Longer intervention durations were associated with larger effect sizes. However, due to small-study effects, limited direct head-to-head comparisons, sparse network connectivity, and the inherent uncertainty of indirect comparisons, the present findings and SUCRA-based rankings should be interpreted with caution.

**Systematic Review Registration:**

https://www.crd.york.ac.uk/PROSPERO/, identifier CRD420261346625.

## 1 Introduction

Developmental Coordination Disorder (DCD) is a prevalent neurodevelopmental disorder, characterized by significant impairments in motor coordination (Biotteau et al., 2020). Its prevalence in children is estimated to be 5%–6% (Gmitrowicz and Kucharska, 1994; Blank et al., 2019). Children with DCD typically exhibit deficits in fine motor skills, hand–eye coordination, and balance (Smits-Engelsman et al., 2013; Kordi et al., 2016; Biotteau et al., 2019; Gao et al., 2025). These impairments negatively impact daily functioning, academic performance, and social participation, and are also closely linked to reduced physical activity, diminished self-efficacy, and various psychological and behavioral issues, such as anxiety and social withdrawal (Cairney et al., 2005a; Engel-Yeger and Hanna Kasis, 2010; Wu et al., 2010; Rivilis et al., 2011; Joshi et al., 2015).

Longitudinal studies have shown that motor deficits in DCD are highly persistent; approximately 50%–70% of affected children continue to face varying degrees of motor difficulties into adolescence, with no natural improvement over time (Tan et al., 2022). This persistent impairment increases the long-term risks of obesity, physical inactivity, and mental health issues (Hinckson et al., 2013; Kuo et al., 2022),Therefore, early intervention is critical, not only for rehabilitation but also for its significant public health value (Caçola, 2016).

Motor interventions are widely used in clinical settings for children with DCD and have been shown to effectively improve motor function (Ferguson et al., 2013; Yu et al., 2018; Peng et al., 2026). These interventions typically include process-oriented training (POT) (Polatajko et al., 1995)and task-oriented training (TOT) (Miyahara et al., 2017). POT focuses on enhancing sensory integration and basic motor control, while TOT emphasizes improving functional performance within specific task contexts (Polatajko et al., 1995; Miyahara et al., 2017). Building on these approaches, integrated training (IT) has emerged as a strategy combining multiple interventions to optimize overall effectiveness (Noordstar et al., 2017).

Despite the growing body of research, significant limitations remain in comparing the relative efficacy of different intervention strategies (Smits-Engelsman et al., 2018; Miyahara et al., 2020). First, many randomized controlled trials (RCTs) compare only one intervention with a control group (CG), lacking direct comparisons between different intervention models (Gao et al., 2025). Second, outcome measures often focus primarily on overall motor skills, with insufficient attention given to key functional domains, such as balance, manual dexterity, and hand–eye coordination (Hirata et al., 2018). Additionally, potential confounding factors, including training methods, dosage, and individual differences, have not been systematically analyzed (Smits-Engelsman et al., 2018). Collectively, these limitations hinder a clear understanding of the relative effectiveness of various intervention strategies.

The core issue in the field is the lack of quantitative evidence that can systematically compare different exercise intervention models within a unified analytical framework, identifying their relative advantages (Salanti, 2012; Puerto Nino and Brignardello-Petersen, 2023). Traditional meta-analyses primarily rely on direct comparisons, making it difficult to integrate multiple interventions and offering no insights into their relative ranking (Lumley, 2002; Catalá-López et al., 2014). In contrast, network meta-analysis integrates both direct and indirect evidence, allowing simultaneous comparisons of multiple interventions and estimation of their relative efficacy, thereby providing higher-level evidence-based support for complex intervention scenarios (Tonin et al., 2017).

To address these gaps, this study incorporated existing evidence from randomized controlled trials and employed a network meta-analysis to compare the effects of POT, TOT, and IT on motor function in children with DCD. The assessment was conducted across four key domains: overall motor ability (Movement Assessment Battery for Children, MABC), balance function, manual dexterity, and aiming and catching ability. This study aims to provide more targeted, evidence-based support for optimizing intervention strategies and informing clinical decision-making for children with DCD.

## 2 Method

This study was conducted and reported in accordance with the Preferred Reporting Items for Systematic Reviews and Meta-Analyses 2020 (PRISMA 2020) statement and its extension for network meta-analyses (PRISMA-NMA) (Hutton et al., 2015; Page et al., 2021). This review was registered in the International Prospective Register of Systematic Reviews (PROSPERO; registration number: CRD420261346625).

### 2.1 Inclusion and exclusion criteria

This study adopted the Population, Intervention, Comparison, Outcome, and Study Design (PICOS) framework to define the inclusion criteria. Studies eligible for this meta-analysis were required to meet the following criteria:

Studies were required to include children diagnosed with DCD. Diagnostic criteria were required to conform to the Diagnostic and Statistical Manual of Mental Disorders, 5th Edition (DSM-5), the International Statistical Classification of Diseases and Related Health Problems, 10th Revision (ICD-10), or international consensus guidelines (e. g., the European guidelines on DCD) (Blank et al., 2019). Alternatively, children diagnosed by healthcare professionals (e. g., pediatricians or physical therapists) via standardized assessment tools, such as the Movement Assessment Battery for Children—Second Edition (MABC-2) (Blank et al., 2019),were also eligible. Participants were required to be aged 5–12 years, with no restrictions on gender or race. This age range was selected because DCD is most commonly identified and diagnosed during early and middle childhood, when motor deficits become evident in academic, play, and daily living activities (Zwicker et al., 2012; Biotteau et al., 2019). In addition, substantial developmental differences in motor competence, physical maturation, and responsiveness to motor interventions may exist between children and adolescents, potentially introducing additional clinical heterogeneity into pooled analyses (Yu et al., 2018; Blank et al., 2019). Although DCD frequently persists into adolescence, the majority of eligible exercise intervention trials identified in the present review were conducted in school-aged children. Therefore, restricting the sample to children aged 5–12 years was intended to improve clinical comparability across studies and reduce age-related heterogeneity.

**Figure 1:**
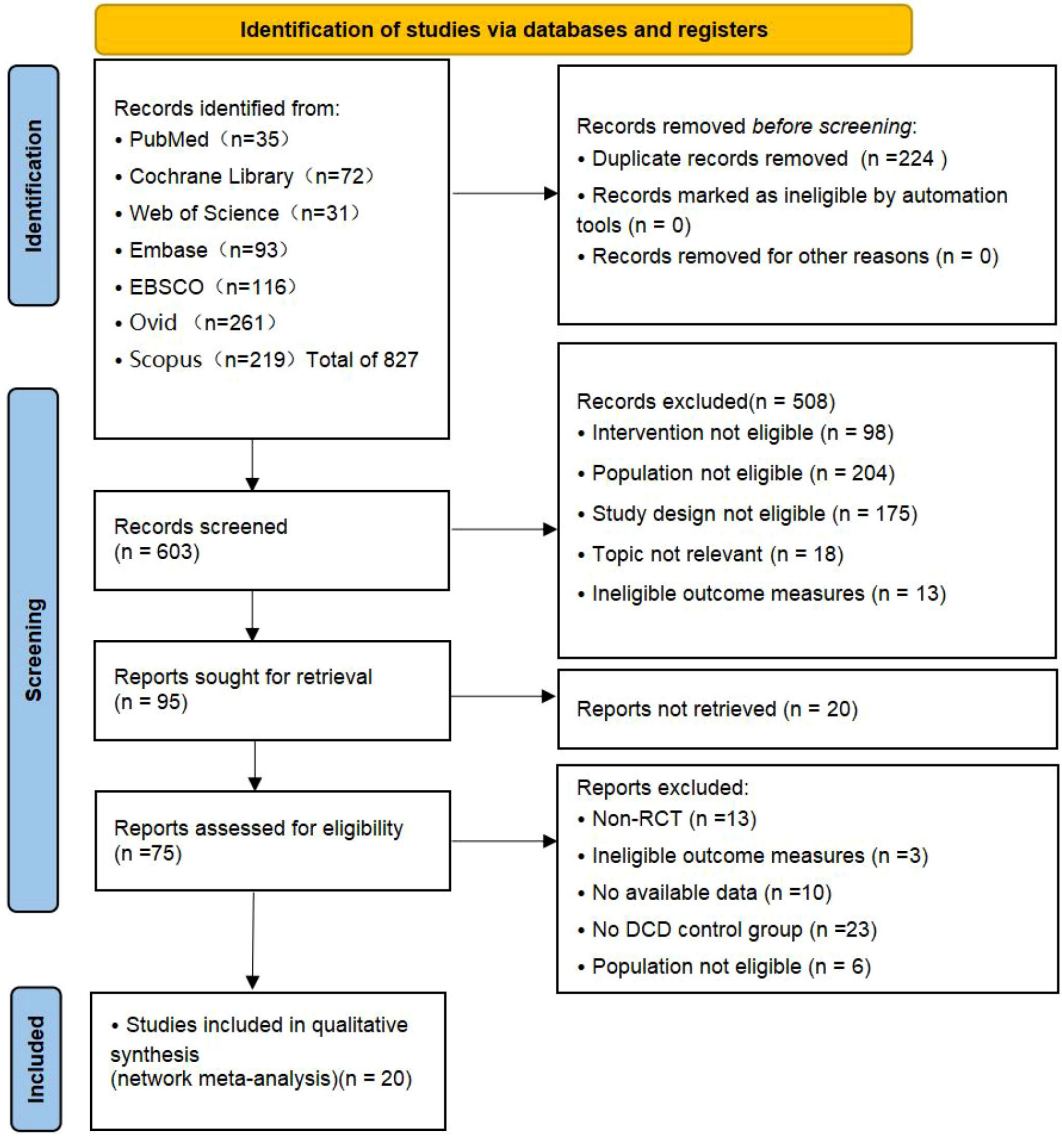
Flowchart for the selection of eligible studies. .

Interventions: Studies were required to include at least one physical activity-based motor intervention, with a clearly described intervention protocol. The specific definitions of these three interventions (IT, TOT, and POT) are presented in **Figure 1**.

Control group: The CG could consist of a different exercise or motor intervention, a non-exercise intervention (e. g., routine care or health education), or a no-intervention/placebo condition.

Outcome Measures: Studies were required to report at least one standardized measure of motor coordination as a primary outcome. Priority was given to studies using the total score or subscales (Balance, Manual Dexterity, and Aiming and Catching) of the Movement Assessment Battery for Children (MABC) or its second edition (MABC-2). Other acceptable outcome measures included the Bruininks–Oseretsky Test of Motor Proficiency, Second Edition (BOT-2), the Test of Gross Motor Development (TGMD), the Körperkoordinationstest für Kinder (KTK), and the Sensory Organization Test (SOT).

Study design: Only RCTs published in peer-reviewed journals were included.

Exclusion Criteria.

Studies were excluded from the analysis if they met any of the following criteria:

1. full text was unavailable;
2. lacked an appropriate control or comparator;
3. had insufficient sample sizes (defined as n < 5 per group) or contained implausible, inconsistent, or duplicate data;
4. contained incomplete data or insufficient information to calculate effect sizes;
5. used outcome measures that did not correspond to the predefined outcomes of this study;
6. focused on other neurodevelopmental disorders (e. g., autism spectrum disorder, cerebral palsy) with DCD as a comorbidity rather than a primary diagnosis.

### 2.2 Interventions

All included motor interventions were categorized into three groups: TOT, POT, and IT. The classification framework was established according to the primary therapeutic mechanism and principal treatment target through which motor improvement was expected to occur, rather than the specific exercise modality itself. This approach was adopted to facilitate meaningful comparisons across heterogeneous intervention protocols within the network meta-analysis, aligning with conceptual frameworks commonly utilized in previous DCD intervention reviews and clinical guidelines (Smits-Engelsman et al., 2013; Smits-Engelsman et al., 2018; Blank et al., 2019).

Because several intervention programs incorporated multiple training components, classification was based on the dominant therapeutic mechanism described by the original authors, the primary intervention objectives, and the main pathway through which motor improvement was intended to be achieved. In brief, the dominant mechanism was independently judged by two reviewers based on the core training goals, the primary therapeutic focus described by the study authors, and the largest proportion of training content explicitly stated in the primary trials, with disagreements resolved through discussion and consensus. When interventions contained multiple training components, classification was determined according to the primary therapeutic focus identified by the original study authors and the principal therapeutic mechanism through which motor improvement was intended to be achieved. Interventions that simultaneously targeted multiple mechanisms without a clearly dominant focus were classified as IT.

TOT was defined as interventions primarily designed to improve motor performance through the direct practice of functional motor tasks in meaningful or ecologically relevant contexts. Representative interventions included NTT (Wilson et al., 2002; Schoemaker et al., 2003; Niemeijer et al., 2006; Winstein et al., 2016),the CO-OP approach (Sugden, 2007; Biotteau et al., 2020), and other task-specific motor learning programs. Although some TOT interventions incorporated cognitive strategies, feedback mechanisms, or problem-solving components, their principal therapeutic target remained the acquisition and refinement of functional motor skills through repeated task practice.

POT was defined as interventions aimed at improving motor performance indirectly by targeting underlying sensorimotor, perceptual, cognitive, or neuromotor processes believed to contribute to motor difficulties in children with DCD. Representative interventions included sensory integration therapy, motor imagery training, visual-perceptual training, action observation training, and related approaches. Because these interventions primarily focused on modifying underlying functional processes rather than practicing target motor tasks directly, they were classified as POT (Sugden, 2007; Biotteau et al., 2020).

IT was defined as interventions that simultaneously incorporated multiple therapeutic components and targeted motor performance through several complementary mechanisms without a single, clearly dominant TOT or POT focus. Representative interventions included taekwondo training, trampoline exercise, cognitive–motor training programs, and balance system training (e.g., iBalance) (Giagazoglou et al., 2015; Fong et al., 2016; Ma et al., 2018; Sun et al., 2021). These interventions typically combined motor skill practice, balance training, coordination exercises, physical conditioning, and cognitive engagement within a unified framework.

Detailed definitions, representative intervention examples, primary therapeutic targets, and study-specific classification rationales are provided in **Table 1** and **Supplementary Table 2**.

**Table 1:** Classification and characteristics of motor intervention types.

Category	Core definition	Key features	Examples
Task-oriented training (TOT)	Focuses on specific functional tasks as tde core of training. It emphasizes repeated practice of motor skills directly related to goal-directed behaviors in real or simulated contexts, to improve task performance capacity.	(1) Training units are task-based; (2) Training content directly corresponds to target functions; (3) Emphasizes task completion and problem-solving skills witdin context.	Aquatic exercise training, cognitive orientation training, virtual reality task-oriented training, Neuromotor Task Training (NTT), Cognitive Orientation to daily Occupational Performance (CO-OP).
Integrated training (IT)	Combines multiple training components (e. g., physical fitness, coordination, and cognitive strategies) through multi-dimensional interventions to work synergistically, aiming to comprehensively improve overall motor function and related abilities.	(1) Diverse intervention content; (2) Involves coordinated training across multiple functional systems; (3) High level of integration among different training components.	Tai Chi combined with strength training, taekwondo, trampoline training combined with cognitive orientation training, iBalance balance training system.
Process-oriented training, (POT)	Targets motor control processes or related foundational functions (e. g., sensory integration, neural processing) as the core of intervention. It aims to indirectly improve motor performance by modifying the underlying mechanisms of motor execution.	(1) Focuses on motor control and sensory processing; (2) Emphasizes improvement of foundational abilities; (3)Training often targets decomposed functional components.	Sensory integration therapy, motor imagery training, visual perceptual training, action observation combined with motor imagery.

Nevertheless, some degree of clinical heterogeneity remained within each category because intervention protocols differed in content, intensity, frequency, implementation procedures, therapist involvement, and training environments. To control the influence of such residual heterogeneity on the pooled results, we preset subgroup analyses by intervention duration and conducted multiple sensitivity analyses excluding outlier studies; the stable outcomes subsequently verified the robustness of our grouping strategy. Therefore, the intervention nodes used in the present network meta-analysis should be interpreted as representing broader rehabilitation approaches rather than completely homogeneous treatment modalities.

Intervention characteristics, including total duration (in weeks), weekly frequency, and session length, were extracted. Based on the duration distribution of published DCD intervention studies (4–18 weeks, with most interventions lasting 8–12 weeks) (Hua et al., 2025), an 8-week cutoff was adopted for subgroup analysis. Eligible studies were divided into two subgroups (≤8 weeks vs. >8 weeks) to explore potential dose–response relationships between intervention duration and therapeutic outcomes.

### 2.3 Retrieval strategies

A comprehensive search strategy was formulated to identify RCTs investigating the effects of motor interventions on motor coordination in children with DCD. Systematic electronic database searches were performed in seven databases: PubMed, the Cochrane Library, Web of Science, Embase, EBSCO, Ovid, and Scopus. The search utilized a combination of Medical Subject Headings (MeSH) and free-text terms, such as “Developmental Coordination Disorder,” “Exercise,” “Child,” “Postural Balance,” and “Randomized Controlled Trial,” with Boolean operators (“AND” and “OR”) to refine the results. The full search strategy is provided in Online Supplementary **Supplementary Table 1**. Additionally, the reference lists of relevant systematic reviews and meta-analyses were manually screened to identify any potentially missing studies not captured in the electronic search. The search was restricted to publications between January 2000 and January 2026.

### 2.4 Filtering and data extraction

Two independent researchers conducted the literature screening and data extraction. Duplicate records were removed using EndNote software (version 21. 0; Clarivate, Philadelphia, PA, USA). Subsequently, the two researchers (Y. D. and R. L.) independently screened potentially relevant studies based on titles and abstracts. Following this step, full texts of potentially eligible studies were retrieved and assessed to determine final inclusion.

Following study selection, data extraction was independently performed by the same two researchers. Discrepancies were resolved through discussion or consultation with a third researcher (X. X.). Extracted data included study characteristics (authors, publication year, region, sample size, participant characteristics), intervention and comparator details, outcome measures, assessment methods, and risk of bias.

Finally, all extracted data were cross-checked by both researchers to ensure accuracy.

### 2.5 Bias risk assessment

Two independent researchers (Y. D. and R. L.) assessed the methodological quality of included RCTs using the Cochrane Risk of Bias 2. 0 (ROB 2. 0) tool, with Review Manager 5. 4 software adopted for auxiliary evaluation (Sterne et al., 2019). Disagreements during the assessment were resolved by group discussion, and a third researcher (X. X.) served as an adjudicator when necessary.

Risk of bias was evaluated across seven predefined domains:

1. Random sequence generation
2. Allocation concealment
3. Blinding of participants and intervention providers
4. Blinding of outcome assessors
5. Completeness of outcome data
6. Selective outcome reporting
7. Other potential sources of bias

Each domain was judged as low risk, unclear risk, or high risk of bias in accordance with the Cochrane Handbook for Systematic Reviews of Interventions. The overall risk of bias was determined based on the following criteria:

1. Overall low risk: All domains were rated as low risk.
2. Overall unclear risk: No high-risk domains were identified, while at least one domain was rated as unclear risk.
3. Overall high risk: At least one domain was judged as high risk.

### 2.6 Confidence in evidence assessment

The confidence of evidence for all network estimates was evaluated using the Confidence in Network Meta-Analysis (CINeMA) framework. CINeMA was applied to assess the certainty of both direct and indirect evidence, as well as the overall network estimates. The framework evaluates six domains, including within-study bias, reporting bias, indirectness, imprecision, heterogeneity, and incoherence. Each comparison was judged as having no concerns, some concerns, or major concerns for each domain. Based on these assessments, the overall confidence of evidence was classified as high, moderate, low, or very low. All evaluations were conducted independently by two reviewers, and any disagreements were resolved through discussion and consensus.

### 2.7 Outcome harmonization

Because different studies adopted diverse validated instruments to assess motor performance, rigorous outcome harmonization was performed prior to quantitative synthesis. Although the MABC, MABC-2, BOT-2, KTK, and independent balance assessments differ in scoring algorithms, item compositions, and original measurement scales, they assess closely related, albeit not identical, domains of pediatric motor competence, including overall motor proficiency, balance control, manual dexterity, and object-control skills (Blank et al., 2019; Huang et al., 2025; Smits-Engelsman et al., 2025). Therefore, outcome pooling in the present study was based on shared motor functional constructs rather than specific assessment instruments. This harmonization strategy has been widely adopted in previous systematic reviews and meta-analyses involving children with DCD, supporting the methodological feasibility of synthesizing functionally comparable outcomes across studies (Miyahara et al., 2017; Smits-Engelsman et al., 2018; Smits-Engelsman et al., 2025).

To account for differences in measurement scales and scoring systems, standardized mean differences (SMDs) were calculated for all pooled analyses. Nevertheless, although SMDs facilitate quantitative synthesis across heterogeneous instruments, they cannot completely eliminate conceptual differences among assessment tools. Consequently, some degree of measurement heterogeneity may remain and should be considered when interpreting pooled effect estimates.

### 2.8 Statistical analysis

Network plots were constructed using the network command in Stata 17. 0, where nodes represented interventions and edges represented direct comparisons. Node size was proportional to the total sample size of each intervention, and edge thickness reflected the number of studies contributing to each comparison. These plots depicted the relationships between direct and indirect evidence across all interventions and control conditions.

Consistency between direct and indirect evidence was assessed using the node-splitting method. A two-sided P < 0. 05 was considered statistically significant. For outcomes with closed evidence loops, inconsistency factors (IFs) and 95% confidence intervals (CIs) were calculated; consistency was confirmed when P > 0. 05. For star-shaped networks without closed loops, formal inconsistency testing could not be performed, as direct and indirect evidence for identical pairwise contrasts cannot be separated statistically.

Given the heterogeneity of measurement tools, pooled effect sizes were estimated as standardized mean differences (SMDs) with 95% CIs. A frequentist random-effects model was applied to synthesize direct and indirect evidence. Forest plots and league tables were constructed for pairwise comparisons, with effect estimates presented as SMDs of row interventions relative to column interventions.

Interventions were ranked using the surface under the cumulative ranking curve (SUCRA) and the probability of being the best (PrBest). Higher SUCRA values indicate superior intervention efficacy.

Subgroup analyses were conducted based on predefined variables: age (≤8 vs. >8 years), intervention duration (≤8 vs. >8 weeks), and study region (Asia, Europe, and Africa). Random-effects models were used for subgroup analyses, and subgroup differences were interpreted qualitatively.

Sensitivity analyses were performed using a leave-one-out approach to quantify the influence of individual studies on overall results. Additional analyses were conducted by excluding studies with extreme effect sizes, identified using influence diagnostics, to assess the robustness of primary findings.

Publication bias and small-study effects were evaluated using Egger’s regression test and funnel plots. A two-sided P < 0. 05 indicated significant small-study effects. Egger’s test was not performed for outcomes with fewer than 10 studies due to insufficient statistical power.

All statistical analyses were performed in Stata 17. 0. Effect sizes were reported as SMDs with 95% CIs. Between-study heterogeneity was assessed using Cochran’s Q test and the I² statistic. An I² > 50% indicated substantial heterogeneity, and a random-effects model was applied in such cases.

### 2.9 Assessment of transitivity

Prior to conducting the network meta-analysis, the transitivity assumption was evaluated by examining the distribution of potential effect modifiers across treatment comparisons. Key clinical and methodological characteristics considered relevant to treatment effects included participant age, baseline motor impairment severity, ADHD comorbidity status, intervention frequency, session duration, total intervention period, and outcome assessment measures. These characteristics were summarized across direct comparison groups (IT vs Control, TOT vs Control, and POT vs Control) and qualitatively compared to identify potential imbalances that could threaten the validity of indirect comparisons. The transitivity assumption was considered plausible when no substantial differences in these effect modifiers were observed across comparison groups.

## 3 Results

### 3.1 Study selection

A total of 827 records were retrieved from comprehensive searches across seven electronic databases: PubMed, the Cochrane Library, Web of Science, Embase, EBSCO, Ovid, and Scopus. After duplicate removal, 603 records remained for title and abstract screening, with 508 records excluded. The remaining 95 articles underwent full-text review.

Full-text screening led to the exclusion of 75 studies: 20 with inaccessible full texts and 55 that did not meet the predefined inclusion criteria. The main reasons for exclusion included: non-randomized controlled trials (n = 13), absence of an appropriate control or comparator for DCD (n = 23), incomplete or non-extractable data (n = 10), ineligible study populations (n = 6), and outcome measures not aligned with the predefined evaluation criteria (n = 3).

Ultimately, 20 studies were included in the network meta-analysis (Pless et al., 2000; Wilson et al., 2002; Hillier et al., 2010; Fong et al., 2012; Coetzee and Pienaar, 2013; Fong et al., 2013; Farhat et al., 2016; Fong et al., 2016; Fong et al., 2016; Kordi et al., 2016; Maharaj and Lallie, 2016; Wilson et al., 2016; Ju et al., 2018; Ma et al., 2018; Marshall et al., 2020; Fong et al., 2022; Babazadeh et al., 2025; Ferreira et al., 2025; Yamanishi et al., 2025; Zhang et al., 2025). **Figure 1** illustrates the overall study selection process.

### 3.2 Study characteristics

A total of 20 RCTs published between 2000 and 2026 were included in the analysis. The studies were conducted across diverse regions, including China (Hong Kong and Taiwan), Australia, Brazil, Iran, Japan, South Africa, Sweden, Tunisia, and the United Kingdom. The total sample size was 1,867 participants, comprising 371 in the integrated training (IT) group, 254 in the task-oriented training (TOT) group, 255 in the process-oriented training (POT) group, and 987 in the CG.

The interventions were categorized into three types. IT (Hillier et al., 2010; Fong et al., 2012; Fong et al., 2013; Maharaj and Lallie, 2016; Ma et al., 2018; Babazadeh et al., 2025; Zhang et al., 2025)encompassed multimodal approaches such as taekwondo training, trampoline exercises combined with CO-OP,Life Kinetic training, and tai chi combined with strength training. OT interventions (Fong et al., 2016; Fong et al., 2016; Biotteau et al., 2020; Ferreira et al., 2025 ,Pless et al., 2000; Farhat et al., 2016)focused on motor skills development through task-specific training, including motor skill training, iBalance balance training, CO-OP cognitive strategy training, aquatic exercise, functional movement training, and group motor skill training. POT interventions (Wilson et al., 2002; Coetzee and Pienaar, 2013; Fong et al., 2016; Kordi et al., 2016; Wilson et al., 2016; Marshall et al., 2020; Fong et al., 2022; Yamanishi et al., 2025)targeted sensorimotor processes, incorporating sensory integration therapy, motor imagery training, muscle strength training, visual perceptual training, and action observation combined with motor imagery.

Outcome measures primarily assessed four functional domains: global motor function, balance, manual dexterity, and aiming and catching ability. Global motor function was assessed using instruments such as the Movement Assessment Battery for Children (MABC/MABC-2), the Bruininks-Oseretsky Test of Motor Proficiency (BOT-2), and the Körperkoordinationstest für Kinder (KTK). Balance function was evaluated using the Sensory Organization Test (SOT), the Modified Clinical Test of Sensory Integration of Balance (mCTSIB), the Unilateral Stance Test (UST), Dynamic Limits of Stability (DLOS), and the Stork Balance Test. Manual dexterity was primarily assessed through tasks related to handwriting, and aiming and catching ability was measured using indicators such as accuracy, reaction time, movement time in eye-hand coordination tasks, and the corresponding subscales of the MABC. The baseline characteristics of the included studies are summarized in **Table 2**.

**Table 2:** Summary of studies included in network meta-analysis.

Study	Country	Participants	Age	Frequency(per week)	Time (weeks)	Duration (mins)	Outcomes
Babazadeh et al. (2025) (Babazadeh et al., 2025)	Iran	IT(20) CG(20)	IT: 10. 73 ± 1. 10 CG: 10. 86 ± 1. 05	2	6	45	KTK RSBT
Coetzee et al. (2013) (Coetzee and Pienaar, 2013)	South Africa	POT(16) CG(16)	–	1	18	40	MABC
Fong^a^ et al. (2016) (Fong et al., 2016)	China	POT (42) TOT (47) CG(41)	FMPT: 7. 8 ± 1. 3 FMT: 7. 9 ± 1. 4 CG: 7. 5 ± 1. 6	2	12	90	SOT
Fongb et al. (2016) (Fong et al., 2016)	China	POT(47) CG(41)	FMT: 7. 9 ± 1. 4 CG: 7. 5 ± 1. 6	2	12	90	MABC
Fongc et sl. (2012) (Fong et al., 2012)	China	IT(21) CG(23)	IT: 7. 7 ± 1. 3 CG: 7. 4 ± 1. 2 TD: 7. 2 ± 1. 0	6	12	60	SOT UST
Fongd et al. (2022) (Fong et al., 2022)	China	IT(TC-MPT)(30) IT(TC) (30) IT(MPT) (30) CG(31)	TC-MPT: 9. 5 ± 1. 1 TC: 9. 9 ± 1. 2 MPT: 9. 8 ± 1. 0 CG: 9. 7 ± 1. 0	2	12	90	MABC-2
Fonge et al. (2013) (Fong et al., 2013)	China	IT (21) DCD (23)	IT: 7. 7 ± 1. 3 CG: 7. 4 ± 1. 2 TD: 7. 2 ± 1. 0	6	12	60	UST MCT
Farha et al. (2016) (Farhat et al., 2016)	Tunisia	TOT(14) CG(13)	TOT(DCD) G: 8. 8 ± 1. 0 CG: 8. 5 ± 0. 6 TD: 8. 6 ± 0. 9	3	8	60	MABC MAT THD Handwriting
Ferreira et al. (2025) (Ferreira et al., 2025)	Japan	TOT(Land)(16) TOT(Water)(19) CG(12)	Land: 7. 6 ± 0. 27 Water: 7. 4 ± 0. 17 CG: 7. 3 ± 0. 32	3	18	60	MABC-2
Hillier et al. (2010) (Hillier et al., 2010)	Australia	TOT(6) CG(6)	TOT: 7. 25 ± 1. 0 CG: 6. 83 ± 1. 2	1	7	30	MABC
Ju et al. (2018) (Ju et al., 2018)	China	TOT(12) CG(12)	5--10	3	4	45	MABC-balance
Kordi et al. (2016) (Kordi et al., 2016)	Iran	POT(15) CG(15)	POT: 8. 01 ± 0. 54 CG: 7. 70 ± 0. 63	2	12	60	BOT-2 balance
Maharaj et al. (2016) (Maharaj and Lallie, 2016)	South African	IT(30) CG(30)	IT: 10. 11 ± 1. 98 CG: 9. 9± 2. 44	1	8	30	MABC Ball skills Balance
Ma et al. (2018) (Ma et al., 2018)	China	IT(51) CG(94)	IT: 6-9	7	12	60	MABC EHC mCTSIB
Marshall et al. (2020) (Marshall et al., 2020)	UK	POT(10) CG(10)	POT: 9. 0 ± 1. 56 CG: 9. 0 ± 1. 41	1	1	60	Target-locking score
Pless et al. (2000) (Pless et al., 2000)	Sweden	TOT(17) CG(20)	TOT: 5. 9 (5. 5-6. 9) CG: 6. 0 (5. 7-6. 7)	1	10	30	MABC
Wilson et al. (2016) (Wilson et al., 2002)	Australia	POT(12) POT(13) CG(11)	7-12	1	5	60	MABC
Wilson et al. (2002) (Wilson et al., 2016)	Australia	POT(PM)(18) POT(C)(18) CG(18)	7-12	1	5	60	MABC
Yamanishi et al. (2025) (Yamanishi et al., 2025)	Japan	POT(9) CG(8)	ASI: 5. 69 ± 1. 34 CG: 5. 83 ± 0. 65	2	10	60	MABC-2
Zhang et al. (2026) (Zhang et al., 2025)	China	IT(10) CG(10)	IT: 5. 25 ± 0. 45 CO-OP: 5. 25± 0. 45 CG-DCD: 5. 20 ± 0. 44	3	12	45	MABC

MABC, Movement Assessment Battery for Children;MABC-2, Movement Assessment Battery for Children-Second Edition;SOT, Sensory Organization Test;UST, Unilateral Stance Test;EHC, Eye-Hand Coordination;mCTSIB, modified Clinical Test of Sensory Integration of Balance;MCT. Motor Control Test;KTK, Körperkoordination Test für Kinder;RSBT, Revised Stork Balance Test;BOT-2, Bruininks-Oseretsky Test of Motor Proficiency Second Edition;TD, Task-oriented Training;MAT. Modified Agility Test;THD. Triple Hop Distance;TC. Tai Chi; MPT. Motor-Perturbation Training. TC-MPT, combined Tai Chi and Motor-Perturbation Training. PM, Psychomotor training. C, Conventional sensory training. ASI, Ayres Sensory Integration. .

### 3.3 Results of risk of bias assessment

The 20 included RCTs demonstrated good methodological quality. Of the studies, 75% were judged to have a low risk of bias, 20% had an unclear risk, and 5% had a high risk of bias. Most trials adequately described the randomization process, while a few failed to report detailed randomization procedures. Regarding allocation concealment, 65% of the studies adopted appropriate measures, while the remaining trials either provided insufficient reporting or lacked effective concealment.

Given the nature of exercise-based interventions, blinding of participants and instructors was not feasible in any of the studies, resulting in a high risk of performance bias. For outcome evaluation, 60% of studies used independent outcome assessors, which helped reduce detection bias. Most trials reported complete outcome data (65%) and demonstrated a low risk of selective reporting (70%), with no significant additional bias identified.

**Figure 2:**
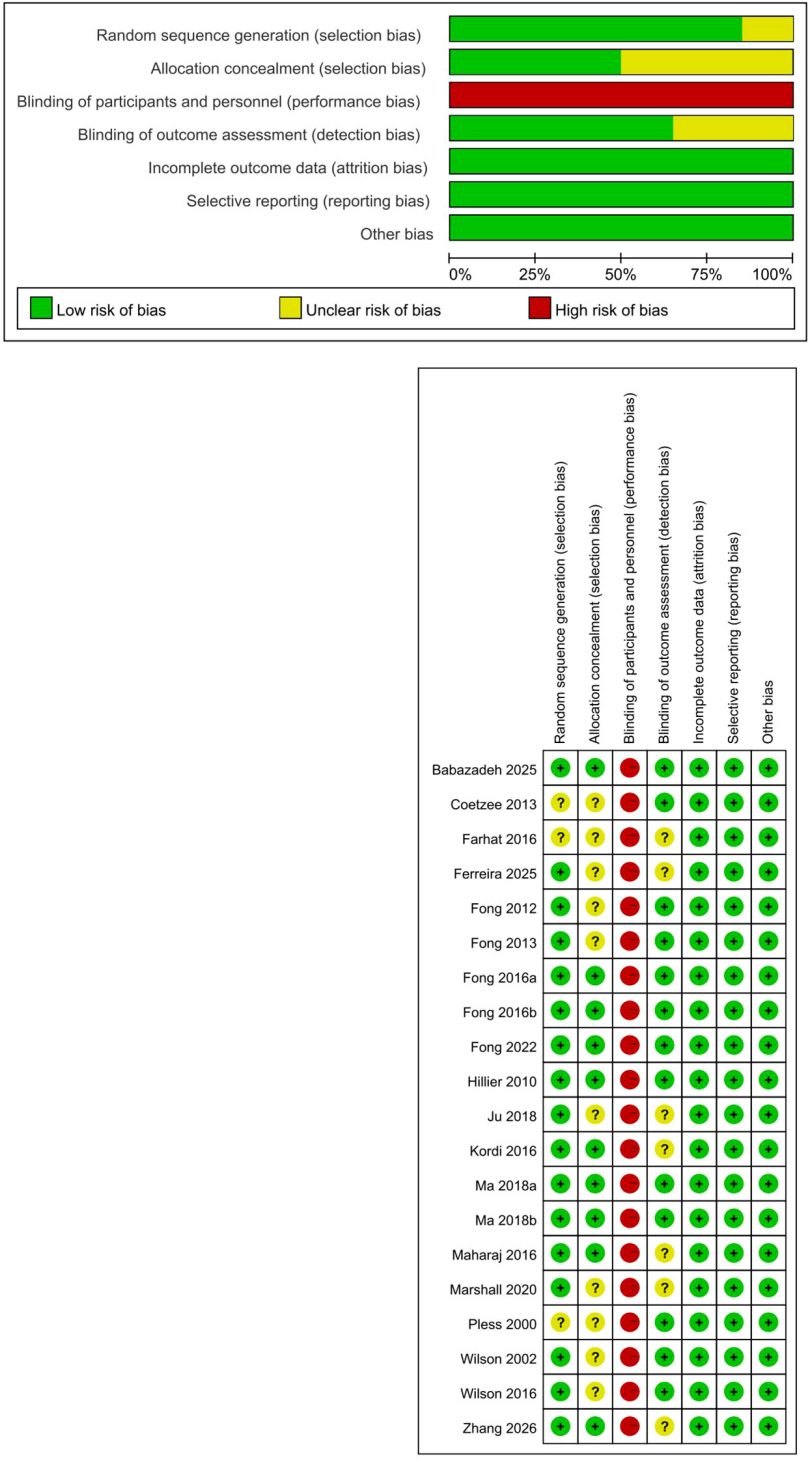
Results of quality assessment of included studies. Quality assessment chart and summary of risk of bias. (Green, yellow and red represent low risk, unclear risk and high risk respectively. Each column represents: randomization process, deviations from intended interventions, missing outcome data, measurement of the outcome, selection of the reported results, and overall bias. ).

Overall, the included RCTs showed good quality in randomization design and outcome reporting. Although inadequate blinding was a major limitation, the overall risk of bias was deemed acceptable for subsequent pooled analysis (**Figure 2**).

### 3.4 Transitivity assessment

The transitivity assumption was evaluated by comparing key clinical and methodological effect modifiers across direct comparison arms (IT vs Control, TOT vs Control, and POT vs Control) (Salanti, 2012; Jansen and Naci, 2013; Salanti et al., 2014). As shown in **Table 3**, participant age (5–12 years), baseline motor impairment severity (predominantly mild-to-moderate DCD), and intervention characteristics (training frequency, session duration, and intervention period) were broadly comparable across studies. All included trials adopted randomized controlled designs and used commonly applied DCD-related outcome measures. Although ADHD comorbidity was inconsistently reported, no evidence suggested systematic differences across comparison groups. Overall, the included studies were considered sufficiently comparable with respect to key effect modifiers, supporting the plausibility of the transitivity assumption and the validity of indirect comparisons within the network.

**Table 3:** Transitivity assessment of included studies grouped by direct comparison arms.

Characteristic	IT vs control (studies, n=7)	TOT vs control (studies, n=5)	POT vs control (studies, n=8)
Clinical characteristics			
Age range (years)	5–12	5–12	6–12
Baseline motor impairment severity	Predominantly mild-to-moderate DCD	Predominantly mild-to-moderate DCD	Predominantly mild-to-moderate DCD
Comorbid ADHD reported	Not reported	Not reported	Not reported
Intervention characteristics			
Intervention frequency (sessions/week)	1–3	1–3	2
Session duration (minutes)	30–60	30–60	40–90
Total intervention period (weeks)	4–18	5–18	5–18
Methodological characteristics			
Primary outcome measures	MABC/MABC-2, BOT-2, SOT, UST	MABC/MABC-2, BOT-2	MABC/MABC-2, SOT
Study design	RCT	RCT	RCT

BOT-2, Bruininks-Oseretsky Test of Motor Proficiency Second Edition;IT, Integrative Training;MABC, Movement Assessment Battery for Children;MABC-2, Movement Assessment Battery for Children-Second Edition;POT, Process-Oriented Training;RCT, Randomized Controlled Trial;SOT, Sensory Organization Test;TOT, Task-Oriented Training;UST. Unilateral Stance Test.

Note. Transitivity was assessed by comparing potential effect modifiers across studies grouped by direct comparison arms (IT vs Control, TOT vs Control, POT vs Control), including participant age, baseline motor impairment severity, ADHD comorbidity status, intervention frequency, session duration, intervention duration, and outcome measures. No major imbalance in these characteristics was observed across comparison groups. Therefore, the transitivity assumption was considered satisfied, supporting the validity of both direct and indirect comparisons within the network. Age range, intervention frequency, session duration, and total intervention period are presented as the range observed across included studies. Predominantly mild-to-moderate DCD was defined based on baseline MABC percentile scores.

### 3.5 Consistency check

Direct and indirect evidence were synthesized to assess comparative intervention effects in children with DCD. **Supplementary Table 3** summarizes the local inconsistency test results for balance, the only outcome with a closed evidence loop (IT–POT–CG).

Node-splitting analysis identified no statistically significant inconsistency. The loop-specific inconsistency factor (IF) was 1. 089 (95% CI: 0. 00, 3. 01, P = 0. 266), with a loop heterogeneity variance (τ²) of 0. 545, suggesting consistent findings between direct and indirect evidence.

**Figure 3:**
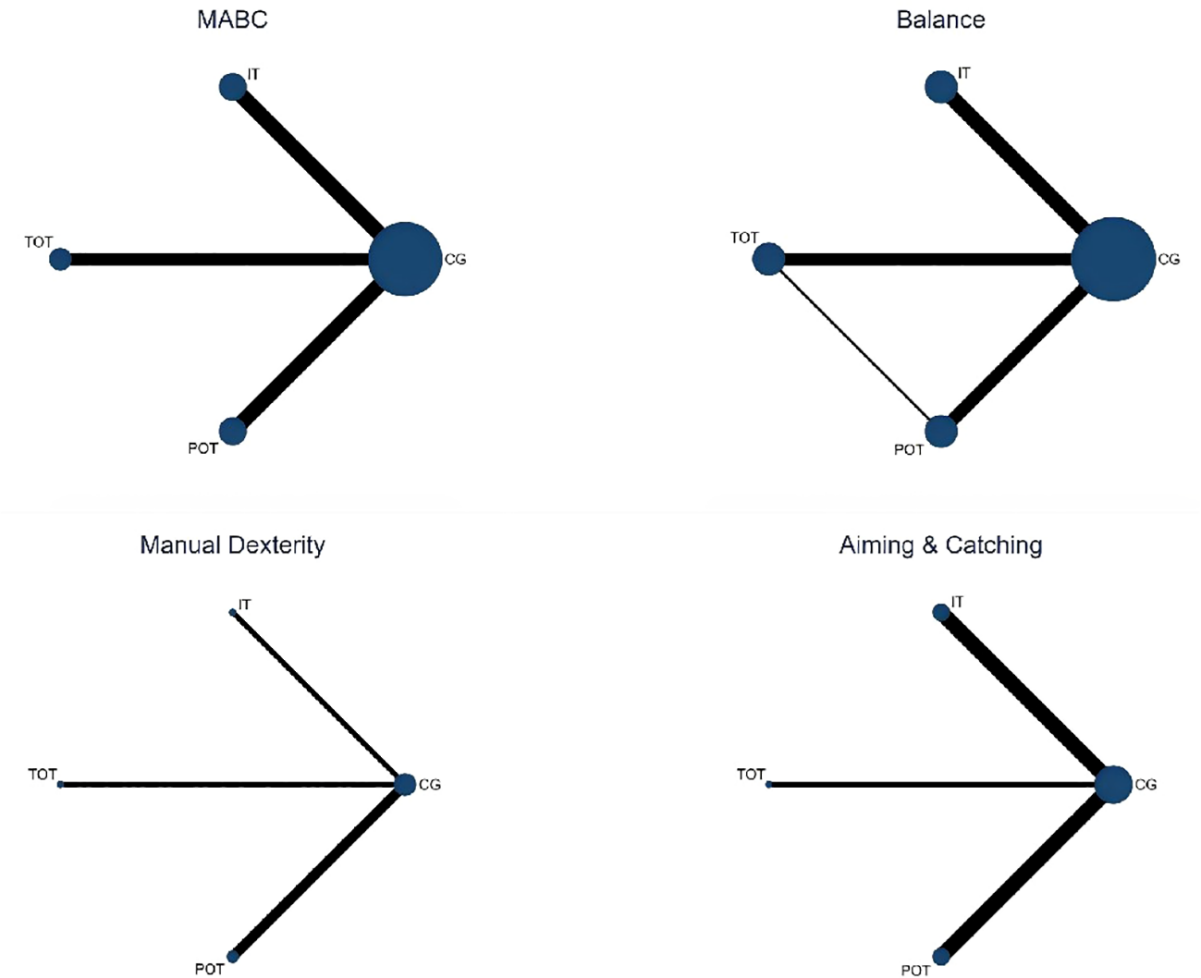
Network diagrams showing direct comparisons among motor interventions across different functional domains.  .

For the remaining outcomes (MABC total score, manual dexterity, and aiming and catching), networks displayed a star-shaped architecture (**Figure 3**): all active interventions were compared solely against the CG, with no head-to-head trials between different active treatments. Owing to the lack of closed loops, formal node-splitting inconsistency assessment was unfeasible, because direct and indirect evidence for the same pairwise comparison could not be statistically separated.

Overall, inconsistency testing was only feasible for balance, for which no inconsistency was observed. These findings supported the consistency assumption, and a consistency-based network meta-analysis model was adopted to calculate pooled effect sizes and intervention rankings.

### 3.6 Results of the network meta-analysis

This study included 20 RCTs involving 1,867 children with DCD, which provided data to compare the efficacy of three motor interventions (IT, TOT, and POT) with a conventional care CG. The consistency assumption was satisfied across all evidence networks, with no significant inconsistencies detected. **Figure 3** illustrates the network evidence structure for four outcome measures: MABC total score, balance function, manual dexterity, and aiming and catching ability. In the network, nodes represent different interventions, and edges indicate direct comparative evidence; node size is proportional to the total sample size of each intervention, while edge thickness reflects the number of studies contributing to each comparison.

**Figure 4:**
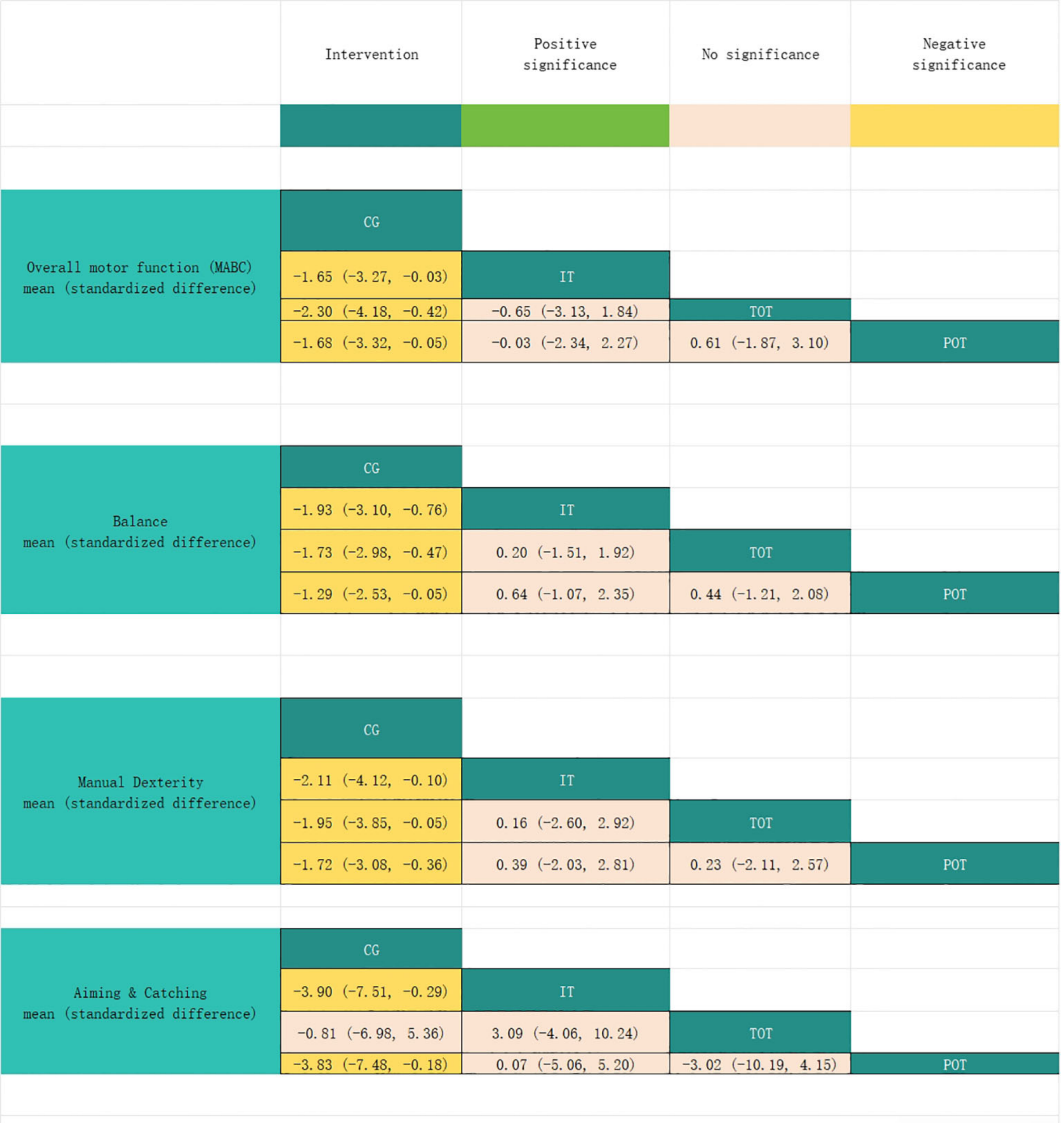
Pairwise comparison effects of motor interventions on functional outcomes in children with developmental coordination disorder. .

**Figure 5:**
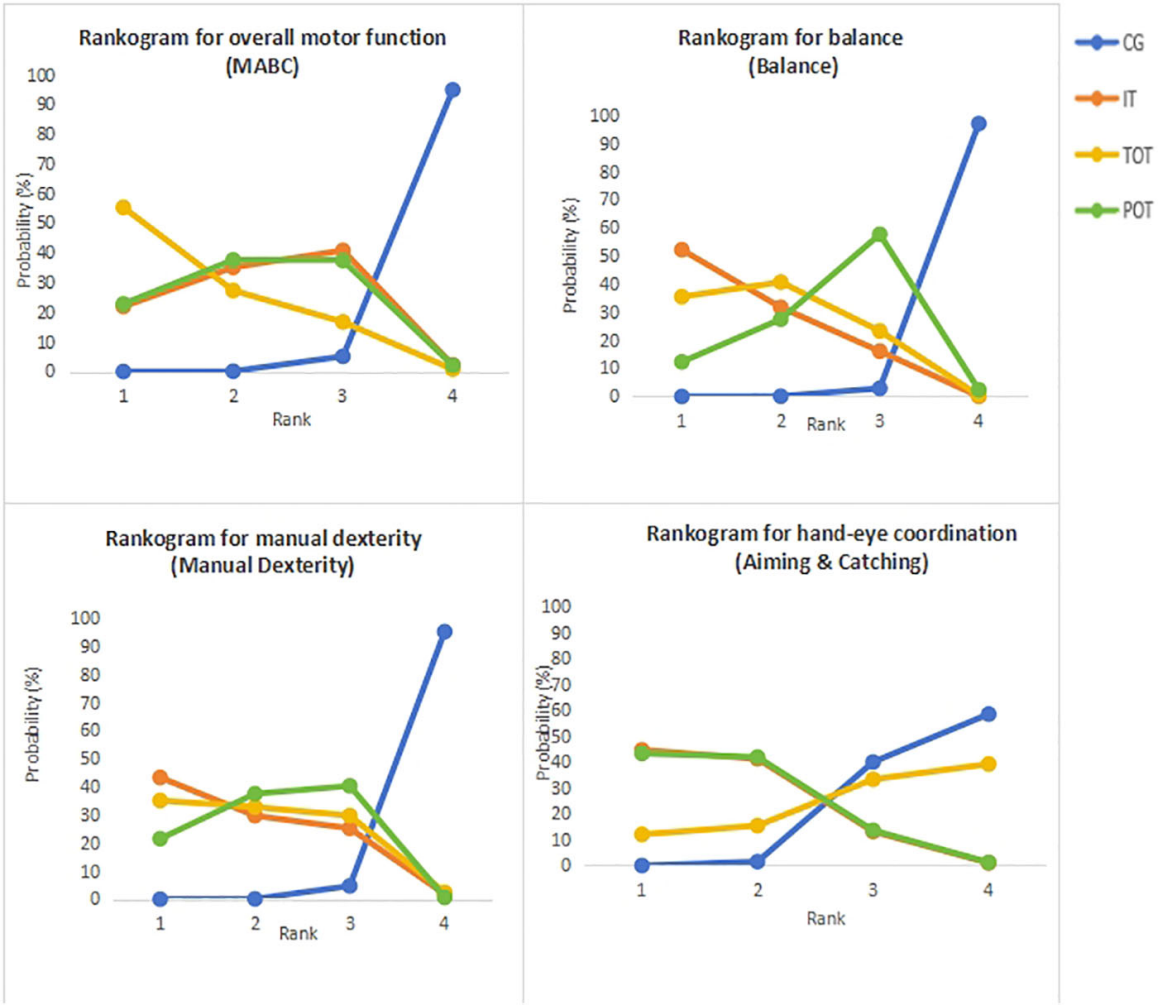
Rankograms for motor interventions across functional outcomes. .

**Figure 4** displays the pooled effect sizes for each intervention from the network meta-analysis. Effect sizes are reported as standardized mean differences (SMDs) with 95% confidence intervals (CIs), where cell shading indicates the statistical significance of treatment effects. **Table 4** and **Figure 5**.

**Table 4:** SUCRA value and rank of interventions.

Outcomes	Treatments	SUCRA	PrBest	Mean rank
MABC	CG	1. 9	0	3. 9
	IT	59. 0	21. 2	2. 2
	TOT	79. 4	56. 2	1. 6
	POT	59. 8	22. 6	2. 2
Balance	CG	0. 8	0	4. 0
	IT	78. 3	51. 7	1. 7
	TOT	70. 4	35. 5	1. 9
	POT	50. 5	12. 8	2. 5
Manual Dexterity	CG	1. 6	0	4. 0
	IT	71. 5	43. 3	1. 9
	TOT	66. 9	35. 1	2. 0
	POT	60. 0	21. 5	2. 2
Aiming and Catching	CG	14. 3	0	3. 6
	IT	76. 6	44. 7	1. 7
	TOT	33. 4	12. 0	3. 0
	POT	75. 7	43. 3	1. 7

MABC, Movement Assessment Battery for Children; CG, usual care or nointervention; IT, Integrative Training; TOT, Task-Oriented Training; POT, Process-Oriented Training.

present the quantitative ranking of intervention efficacy based on surface under the cumulative ranking curve (SUCRA) values.

The network meta-analysis results indicated outcome-specific efficacy differences among the three interventions across the four domains evaluated.

#### 3.6.1 MABC

**Figure 6:**
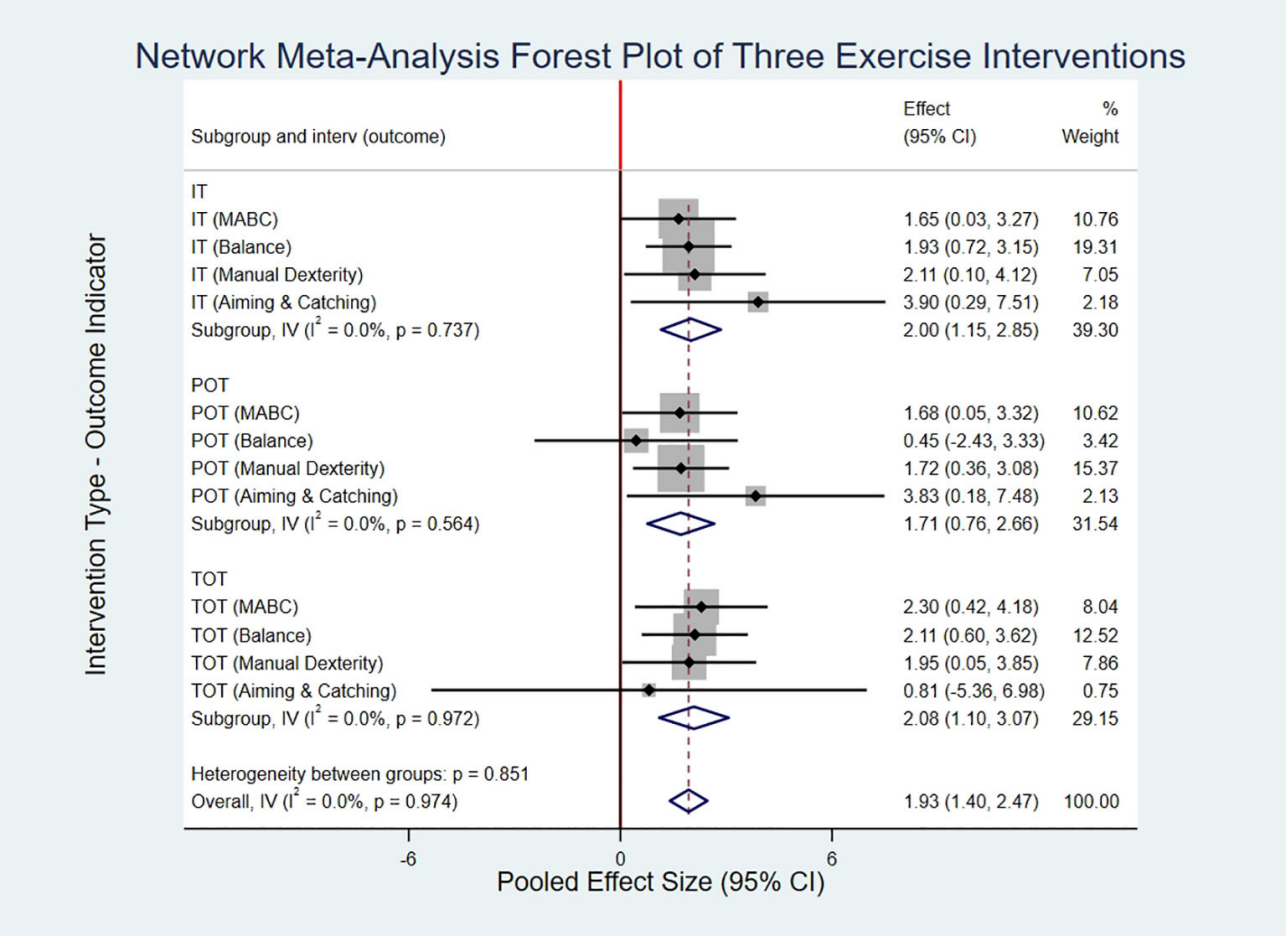
Network meta-analysis forest plot.

Fourteen randomized controlled trials involving 624 children with DCD were included for analysis of the MABC total score. All three motor interventions (IT, TOT, and POT) demonstrated significant improvements in overall motor function compared with the CG: TOT (SMD = 2. 30, 95% CI: 0. 42, 4. 18), IT (SMD = 1. 93, 95% CI: 0. 72, 3. 15), and POT (SMD = 1. 68, 95% CI: 0. 05, 3. 32) (**Figure 6**). Based on SUCRA values, TOT ranked highest in improving overall motor function (SUCRA = 55. 3%) (**Table 4**).

#### 3.6.2 Balance

The analysis included 15 studies involving 786 children with DCD, assessing the effects of three exercise interventions (IT, TOT, and POT) and a CG on balance function. **Figure 6** shows that both IT (SMD = -1. 93, 95% CI: -3. 15, -0. 72) and TOT (SMD = -2. 11, 95% CI: -3. 62, -0. 60) significantly improved balance function compared with the CG, while POT was not statistically significant (SMD = -0. 45, 95% CI: -3. 33, 2. 43). SUCRA ranking from network meta-analysis indicated that IT yielded the best efficacy for balance improvement, with a SUCRA value of 78. 3% (**Table 4**).

#### 3.6.3 Manual dexterity

The network meta-analysis of manual dexterity included four studies involving 96 children with DCD, assessing the effects of three exercise interventions (IT, TOT, and POT) and a CG. All three interventions significantly improved manual dexterity compared to the CG. The effect sizes were as follows: IT (SMD = -2. 11, 95% CI: -4. 12, -0. 10), TOT (SMD = -1. 95, 95% CI: -3. 85, -0. 05), and POT (SMD = -1. 72, 95% CI: -3. 08, -0. 36) (**Figure 6**). SUCRA ranking from the network meta-analysis revealed that IT had the highest probability of superior efficacy for manual dexterity (SUCRA = 71. 5%, **Table 4**).

#### 3.6.4 Aiming and catching

A network meta-analysis evaluating aiming and catching ability included seven studies with 320 children diagnosed with DCD, comparing three motor interventions (IT, TOT and POT) with a CG. The results indicated that both IT (SMD = -3. 90, 95% CI: -7. 51, -0. 29) and POT (SMD = -3. 83, 95% CI: -7. 48, -0. 18) significantly improved aiming and catching ability compared with the control group, whereas TOT showed no significant effect (SMD = -0. 81, 95% CI: -6. 98, 5. 36) (**Figure 6**). SUCRA rankings from the network meta-analysis indicated that IT and POT had the highest probability of being the most effective for improving aiming and catching ability (SUCRA = 76. 6% and 75. 7%, respectively; **Table 4**).

#### 3.6.5 Subgroup analysis

To explore potential sources of heterogeneity among the included studies, subgroup analyses were conducted based on three categorical variables: participant age (≤8 years, >8 years), intervention duration (≤8 weeks, >8 weeks), and study region (Asia, Europe, Africa). In the age subgroup, the pooled effect size for children older than 8 years (SMD = 1. 96) was slightly higher than that for children aged 8 years or younger (SMD = 1. 60); however, no statistically significant between-group difference was observed (P = 0. 577). Both age groups demonstrated significant benefits from exercise interventions. For intervention duration, the long-term intervention group (>8 weeks) showed a significantly larger effect size (SMD = 1. 95) compared with the short-term group (≤8 weeks) (SMD = 1. 30, P = 0. 001), suggesting that longer intervention duration may be associated with greater therapeutic effects. Regarding study region, the largest effect was observed in Africa (SMD = 3. 98), followed by Asia (SMD = 2. 41) and Europe (SMD = 1. 95), with statistically significant between-region differences (P = 0. 001). However, as only three studies were conducted in Africa, these findings should be interpreted with caution. These differences may be attributed to variations in baseline physical activity levels and intervention standardization across regions. Intervention rankings across subgroups were consistent with those of the primary analysis and did not alter the overall conclusions, supporting the robustness of the main findings. Detailed subgroup results are presented in **Table 5**.

**Table 5:** Subgroup analysis of pooled SMD for motor function in children with DCD.

Subgroup	Subgroup category	N	Z	SMD	95% CI	I²(%)	P	Weight (%)
Age	≤8 years	7	3. 748	1. 6	[0. 76,2. 44]	84. 9	<0. 001	49. 57
	>8 years	7	4. 029	1. 96	[1. 01,2. 92]	92. 7	<0. 001	50. 43
Test for subgroup differences: Chi² = 0. 31, P = 0. 577, I² = 0%								
Duration of intervention	Short-term	9	7. 64	1. 3	[0. 97,1. 62]	85. 1	<0. 001	65. 95
	Long-term	9	9. 74	1. 95	[1. 57,2. 34]	92. 7	<0. 001	34. 05
Test for subgroup differences: Chi² = 11. 20, P = 0. 001, I² = 91. 1%								
Country	Asia	11	5. 33	2. 41	[1. 50,3. 32]	79. 2	<0. 001	39. 8
	Africa	3	6. 13	3. 98	[2. 76,5. 21]	0	<0. 001	17. 6
	Europe	4	7. 64	1. 95	[1. 57,2. 34]	0	<0. 001	42. 6
Test for subgroup differences: Chi² = 15. 50, P = 0. 001, I² = 87. 1%								

N, number of included studies; Z, z-statistic; SMD, standardized mean difference; CI, confidence interval; I², heterogeneity statistic.

#### 3.6.6 Sensitivity analysis

To assess the robustness of the pooled results, two sensitivity analyses were performed across all outcome measures. First, a leave-one-out sensitivity analysis was conducted by omitting each study in turn and recalculating the pooled effect size. The results showed that the pooled estimates remained stable, ranging from 1. 80 to 2. 01, and all corresponding 95% confidence intervals did not include zero. No single study exerted a substantial influence on the overall effect size, indicating the robustness of the findings (**Supplementary Table 6**). Second, after excluding studies with extreme effect sizes, the pooled effect size remained statistically significant (SMD = 1. 95, 95% CI: 1. 57, 2. 34). These findings further confirm the stability and reliability of the primary results. Notably, several included studies were judged as having high or unclear risk of bias, especially regarding blinding of participants and outcome assessors. Given the practical characteristics of motor rehabilitation training, full participant blinding is nearly unachievable in pediatric intervention trials, which may trigger performance and detection bias and potentially inflate pooled intervention efficacy. Formal sensitivity analysis by excluding all high/unclear-bias studies was not feasible: removal of these trials would drastically reduce the number of available comparisons, destroy the integrity of existing evidence networks and invalidate subsequent network meta-analysis. Therefore, we acknowledge the possible overestimation of true therapeutic efficacy originating from unblinded, high-risk trials, and readers are recommended to interpret the pooled positive effects cautiously considering this inherent methodological limitation.

#### 3.6.7 Assessment of publication bias

Publication bias was assessed using Egger’s regression test combined with a corrected funnel plot. The results indicated significant small-study effects for the MABC outcome (intercept = 4. 95, 95% CI: 2. 29, 7. 62, P = 0. 002), the balance function outcome (intercept = 6. 88, 95% CI: 4. 40, 9. 36, P < 0. 001), and the aiming and catching outcome (intercept = 6. 87, 95% CI: 2. 71, 11. 03, P = 0. 008). For the manual dexterity outcome, only four eligible studies were included. Due to the limited number of studies, Egger’s test was not statistically significant (P = 0. 745), and a reliable assessment of publication bias was not feasible. Overall, except for manual dexterity, which could not be reliably evaluated due to insufficient studies, the other three outcomes showed significant small-study effects. These findings suggest that the pooled effect sizes should be interpreted with caution (**Supplementary Figure 1**; **Supplementary Table 7**).

#### 3.6.8 Confidence in evidence assessment

The confidence of network estimates was assessed using the CINeMA framework (**Supplementary Table 8**). Across all outcomes, confidence ratings ranged from low to moderate. For MABC, most comparisons were rated as low confidence owing primarily to heterogeneity and imprecision. Similar patterns were observed for balance and manual dexterity outcomes. In the aiming and catching network, confidence ratings were generally low to moderate, although concerns regarding within-study bias, reporting bias, and imprecision remained. Overall, heterogeneity, imprecision, reporting bias, and residual measurement heterogeneity arising from the use of different motor assessment instruments were the most common factors contributing to the downgrading of evidence confidence.

## 4 Discussion

This study conducted a network meta-analysis to systematically evaluate the effects of different motor intervention strategies on motor function among children with DCD, and to compare the relative efficacy of various interventions across multiple outcome indicators within a unified analytical framework. A total of 20 randomized controlled trials involving 1,867 participants were included. Four core outcomes were focused on, including MABC, balance, manual dexterity, and aiming and catching ability, so as to clarify the relative advantages of different intervention protocols. The results showed that IT, TOT and POT all significantly improved motor function in children with DCD, whereas their efficacy varied distinctly across different outcomes. In terms of overall motor function (MABC), TOT yielded the largest effect size and the highest SUCRA ranking. For balance function, IT exerted the optimal intervention effect. For manual dexterity, IT also presented the highest superiority probability. Regarding aiming and catching ability, both IT and POT showed prominent therapeutic effects with comparable superiority probabilities. Of note, all SUCRA rankings represent probabilistic comparisons rather than definitive optimal results, given sparse head-to-head trials and limited available evidence across most endpoints.

### 4.1 MABC

The present study identified that TOT presented unique advantages in improving the overall motor function of children with DCD. Previous evidence has demonstrated that functional task-centered interventions can effectively enhance general motor performance and its subdomains, including fine motor skills and hand–eye coordination (Au et al., 2014; Miyahara et al., 2017; Peng et al., 2026). Relevant randomized controlled trials have further supported this viewpoint (Wilson et al., 2016).

This superior efficacy is largely explained by task-specific learning mechanisms (Miyahara et al., 2017). Children with DCD typically exhibit prominent deficits in procedural learning (Al-Yahya et al., 2023; Van Dyck et al., 2023). As a targeted training mode, TOT embedded repetitive practice in daily functional scenarios such as writing and ball games, which strengthened the consolidation of stable motor patterns and facilitated skill transfer to daily living activities (Schoemaker et al., 2003). Moreover, training highlighting active participation and problem-solving strategies could enhance intervention adherence, thereby promoting the acquisition and long-term retention of motor skills (Farhat et al., 2016).

### 4.2 Balance

Balance control depends on the coordinated regulation of proprioceptive and vestibular inputs, as well as the postural control system. Accordingly, single-modal training often fails to improve balance function comprehensively (Allum et al., 1998; Cyr et al., 2019). As an integrated intervention, IT combines strength training, functional movement and sensory stimulation, targeting proprioception, muscle strength and postural regulation simultaneously. Such systematic training mode effectively facilitates balance improvement in children with DCD (Winter et al., 2022).

Consistent with the above mechanism, the present findings confirmed that IT produced the optimal effects on balance. Previous meta-analyses and systematic reviews have also demonstrated that combined interventions integrating task-oriented and process-oriented training are superior to single-mode programs (Smits-Engelsman et al., 2013; Gao et al., 2025; Peng et al., 2026),Relevant randomized controlled trials have likewise reported significant balance gains after comprehensive exercise training (Fong et al., 2016; Fong et al., 2016). Furthermore, embedding training within functional scenarios can enhance sensorimotor integration and optimize dynamic postural control (Fong et al., 2016). Meanwhile, diversified training contents enrich participants’ experience, improve long-term adherence, and thus further boost overall intervention effectiveness (Collado-Mateo et al., 2021; André et al., 2024).

### 4.3 Manual dexterity

For manual dexterity, IT also presented a distinct comparative advantage. Unlike gross motor performance, fine motor tasks require high-precision sensorimotor integration, while single-modal training hardly meets the dual demands of sensory input and precise motor control simultaneously (Lin et al., 2024).

By integrating sensory integration training with functional tasks, IT delivered multidimensional sensory and motor stimulation to support fine motor regulation (Case-Smith, 1995). On the one hand, multimodal training enhances proprioceptive perception and consolidates fundamental motor control ability (Lim, 2019; Winter et al., 2022). On the other hand, embedded functional tasks promote the transfer of training gains to daily fine motor behaviors, such as grasping and object manipulation (Schaefer et al., 2013; Ma et al., 2018). Furthermore, diversified training modes sustain children’s active engagement, which further improves intervention efficiency. Relevant randomized controlled findings also partly corroborate the above mechanism (Legarra-Gorgoñon et al., 2025).

### 4.4 Aiming and catching

Different from other indicators, both IT and POT yielded significant improvements in aiming and catching ability, while TOT showed no obvious therapeutic advantage. Compared with general motor functions, aiming and catching rely more on precise coordination among visual processing, proprioceptive feedback and upper-limb motor control, which requires efficient sensory integration and rapid motor adjustment. Thus, the intervention mechanisms for this skill are relatively complex (Hagura et al., 2009).

Aiming and catching performance depends on the coordinated interaction of vision, proprioception and motor regulation, in which basic motor capacities play a critical supporting role (Dessing et al., 2009). POT strengthens sensory integration and fundamental motor control, laying a solid foundation for the acquisition of complex motor skills (Chen et al., 2020). As a multimodal intervention, IT delivers comprehensive sensory and motor stimulation to optimize integrated regulatory processes, thereby improving complex motor performance (Lim, 2019). In comparison, TOT focuses heavily on repeated training of single functional tasks. Its efficacy is highly context-dependent, with limited cross-task transferability, which restricts its benefits for compound skills such as aiming and catching (Schaefer et al., 2013).

Although IT and POT demonstrated large effect sizes for aiming and catching performance, these findings should be interpreted with caution. The observed SMD values approached or exceeded 3. 0, which are considerably larger than those commonly reported in pediatric rehabilitation research. Several factors may have contributed to these unusually large estimates. First, only a limited number of studies were available for this outcome, resulting in reduced statistical precision and increased susceptibility to small-study effects. Second, substantial differences existed across studies in intervention protocols, outcome assessment procedures, and participant characteristics, which may have amplified between-study variability and inflated standardized effect estimates. Third, publication bias cannot be excluded, as studies reporting positive findings are more likely to be published. Therefore, although the results suggest potential benefits of IT and POT for improving aiming and catching performance, the magnitude of these effects should not be interpreted as definitive evidence of superior clinical efficacy. Additional large-scale and well-controlled head-to-head trials are required to verify these findings.

### 4.5 Efficacy of the intervention and its determinants

All subgroup analyses conducted in this study should be regarded as exploratory and were primarily performed to identify potential sources of between-study heterogeneity rather than to establish definitive subgroup-specific effects. Given the limited number of studies within several subgroup categories, particularly in regional comparisons, the corresponding estimates may be unstable and susceptible to random variation. Therefore, these findings should be interpreted cautiously and should not be considered conclusive evidence of true subgroup differences.

Subgroup analysis indicated that interventions lasting more than 8 weeks produced a higher effect size than those with a shorter duration (≤8 weeks), which was consistent with the primary analysis and confirmed that intervention duration was a key determinant of therapeutic efficacy. Although both short-term and long-term protocols yielded favorable effects, prolonged intervention contributed to additional functional benefits, which provides valuable reference for clinical practice.

This difference conformed to the developmental characteristics of children with DCD, who require frequent and repetitive training to acquire stable motor skills (Hyde and Wilson, 2011; Smits-Engelsman et al., 2018). Nevertheless, previous meta-analyses proposed that improvements in fine motor performance may be inversely associated with training dose, indicating a potential dose-threshold effect (Peng et al., 2026). In accordance with the present results, simply extending intervention duration cannot guarantee continuous incremental benefits. Excessively intensive training may increase physical load and induce fatigue, thereby weakening intervention effects (Sweller, 1988). Meanwhile, relevant indicators regarding training standardization and participant adherence were not measured in the current study, which cannot be excluded as confounding factors. Therefore, reasonable intervention planning should focus on sufficient rather than excessive training volume.

In terms of age subgroups, children aged over 8 years presented a marginally higher effect size than younger participants (≤8 years), while the between-group difference lacked statistical significance. It further demonstrated that exercise interventions exerted favorable effects across all age groups of children with DCD. This trend was consistent with the primary analysis and supported by previous research (Farhat et al., 2016). Relevant evidence focusing on aquatic exercise also confirmed the benefits of early intervention among DCD children aged 6–10 years (Hillier et al., 2010). Given the non-significant inter-age difference and limited subgroup sample size, these age-related findings should be interpreted cautiously.

Regional subgroup analysis revealed that African studies reported the largest intervention effect, which was significantly higher than that in Asian and European cohorts. However, this conclusion was based on a small number of regional studies, and the extreme effect value implied a high risk of overestimation. Such regional discrepancies may stem from differences in children’s baseline physical fitness and intervention implementation conditions (Naylor and McKay, 2009). Furthermore, unbalanced regional study distribution and potential publication bias also interfered with the pooled results. Hence, multi-region and large-sample studies are still required to verify these regional differences.

### 4.6 Robustness of results and methodological considerations

Sensitivity analyses yielded highly consistent findings, and no individual study exerted a dominant influence on the pooled estimates. Multiple analytical strategies were performed, including leave-one-out analyses, exclusion of outliers, adjustment of statistical models, and removal of small-sample studies. Although minor fluctuations in effect sizes were observed across analyses, the overall direction and statistical significance of the primary outcomes remained unchanged. These findings support the robustness of the present results under different analytical assumptions.

Nevertheless, some degree of between-study heterogeneity remained, potentially arising from differences in intervention frameworks, training contents, and outcome assessment tools across trials (Hillier et al., 2010; Biotteau et al., 2019). For instance, fundamental differences in task design and intervention objectives between TOT and POT further diversified intervention effects. Subgroup analyses further verified that intervention duration and regional factors partly accounted for outcome discrepancies.

Moreover, although outcomes were harmonized by grouping assessments according to shared motor functional domains, inherent conceptual and methodological differences across measurement instruments should be acknowledged. Widely adopted tools such as the MABC, BOT-2, and KTK capture overlapping but non-identical aspects of motor competence and employ different scoring systems and evaluation frameworks (Blank et al., 2012; Lucas et al., 2013; Smits-Engelsman et al., 2013). Consequently, residual measurement heterogeneity may have reduced cross-study comparability and contributed to variability in pooled effect estimates, despite the use of outcome harmonization procedures and standardized mean differences.

Notably, such heterogeneity did not alter the core conclusions of the present study. Intervention rankings remained stable across all sensitivity tests, and network consistency diagnostics showed no significant inconsistency. Therefore, the overall network structure and heterogeneity level were within a reasonable and acceptable range.

In terms of methodological quality, most included studies met basic research standards and followed standardized randomization and reporting norms. However, inadequate blinding was a common limitation in exercise intervention trials. Lack of assessor blinding may introduce subjective bias to judgment-dependent outcomes and increase the risk of effect overestimation. Although several studies reduced detection bias by adopting independent evaluators, such confounding bias could not be fully eliminated. Hence, the interpretation of effect sizes should be treated with caution. Overall, the current evidence presented acceptable methodological reliability, and higher-quality controlled trials are still required to further validate these conclusions.

### 4.7 Risk of bias and certainty of evidence

Egger’s regression tests revealed significant small-study effects for MABC, balance, and aiming and catching outcomes. Manual dexterity could not be formally assessed because of the limited number of eligible studies. These findings suggest the possible presence of publication bias and selective reporting, as studies with non-significant or unfavorable results may remain unpublished. Accordingly, pooled effect estimates and intervention rankings may have been inflated.

The unusually large effect sizes observed for several outcomes also warrant cautious interpretation. In particular, the pooled effects for aiming and catching reached or exceeded an SMD of 3. 0, a value substantially higher than those commonly reported in pediatric rehabilitation research. Such extreme estimates may partly reflect small-study effects, publication bias, limited sample sizes, considerable between-study variability, and sparse evidence networks, and therefore should be interpreted with caution.

From a network perspective, all outcomes except balance presented predominantly star-shaped network structures, with very few direct head-to-head comparisons among IT, TOT, and POT. As a result, most comparative estimates relied heavily on indirect evidence. Although no statistically significant inconsistency was detected, the limited availability of direct evidence and sparse network connectivity inevitably reduce confidence in comparative efficacy estimates and treatment rankings.

Furthermore, certainty of evidence was evaluated using the CINeMA framework. Across all outcomes, confidence ratings ranged from low to moderate. Heterogeneity, imprecision, reporting bias, and reliance on indirect comparisons were the primary factors contributing to the downgrading of evidence certainty. These findings indicate that, despite statistically significant intervention effects and relatively stable ranking patterns, the overall certainty supporting comparative efficacy estimates remains limited. Therefore, SUCRA-based rankings should be interpreted as probabilistic estimates of relative treatment performance rather than definitive evidence of intervention superiority.

### 4.8 Clinical implications and limitations of the study

The present findings suggest that different motor intervention strategies may provide distinct benefits across functional domains in children with DCD. Specifically, TOT appeared to be more beneficial for overall motor function, whereas IT demonstrated favorable effects on balance and manual dexterity. Both IT and POT showed potential advantages for complex motor skills such as aiming and catching. These findings may assist clinicians in selecting intervention strategies according to specific functional goals and individual rehabilitation needs (Naylor and McKay, 2009). Individualized intervention schedules can be further tailored for different pediatric populations. For example, shorter and more frequent training sessions may be more suitable for younger children, whereas supplementary multisensory approaches may benefit children with DCD who present with attentional difficulties (Gao et al., 2024). Nevertheless, these clinical recommendations should be considered preliminary and require further empirical validation.

Several limitations of this study should be acknowledged.

First, although outcome harmonization enabled quantitative synthesis across studies using different assessment instruments, conceptual and methodological differences among motor performance measures may have introduced residual measurement heterogeneity. Instruments such as the MABC, BOT-2, and KTK assess overlapping but not identical constructs and differ in scoring frameworks and psychometric properties. Although standardized mean differences were used to facilitate comparisons across instruments, this approach cannot completely eliminate differences in measurement characteristics. Therefore, pooled effect sizes should be interpreted as estimates of relative improvements within broadly comparable motor domains rather than precise measures of identical clinical constructs across assessment instruments.

Second, the present review was restricted to children aged 5–12 years, which may limit the generalizability of the findings to adolescent populations with DCD. Given the substantial developmental changes in motor proficiency, physical maturation, cognitive function, and social participation that occur during adolescence, the effectiveness and responsiveness to exercise interventions may differ from those observed in younger children. Moreover, DCD-related motor difficulties often persist into adolescence, where functional demands and participation contexts become increasingly complex. Therefore, the present findings should be interpreted primarily within the context of school-aged children, and future research is needed to determine whether similar comparative effects are observed among adolescents with DCD (Bonney et al., 2017; Miyahara et al., 2017).

Third, most included studies evaluated only short-term intervention outcomes, and long-term follow-up data were scarce. Consequently, the sustainability of intervention-induced improvements and their long-term influence on functional participation remain uncertain.

Fourth, several clinically important moderators, including baseline motor impairment severity, comorbid conditions, intervention adherence, family environmental factors, and sex-related differences, could not be comprehensively examined because of inconsistent reporting across primary studies (Blank et al., 2019; Smits-Engelsman et al., 2022; Gao et al., 2024). In particular, sex-stratified subgroup analyses were not feasible because most included studies reported only aggregated outcomes without providing separate results for boys and girls. Despite the higher prevalence of DCD among boys (Blank et al., 2019; Li et al., 2024), the potential moderating effect of sex on intervention efficacy remains unclear and warrants further investigation. Future studies should report sex-specific outcomes to facilitate subgroup analyses and improve understanding of potential sex-related differences in intervention responsiveness.

Fifth, from a network structural perspective, the evidence networks for MABC total score, manual dexterity, and aiming and catching were predominantly star-shaped without closed evidence loops, which precluded formal node-splitting inconsistency testing for these outcomes. Although no significant inconsistency was identified via node-splitting analysis for balance (the only outcome with available closed loops), the overall scarcity of direct head-to-head comparisons among active interventions represents an important methodological limitation. Consequently, most comparative estimates relied heavily on indirect evidence, which inevitably reduces confidence in comparative efficacy estimates and treatment rankings despite the absence of statistically significant inconsistency.

Sixth, certainty of evidence was generally rated as low to moderate according to the CINeMA framework. Heterogeneity, imprecision, reporting bias, and reliance on indirect evidence were the primary factors contributing to the downgrading of evidence certainty. Therefore, SUCRA-based rankings should be interpreted as probabilistic estimates of relative treatment performance rather than definitive evidence of intervention superiority.

Seventh, Despite the predefined classification framework, some interventions contained overlapping characteristics and could plausibly be assigned to more than one category. Consequently, the intervention nodes should be interpreted as representing broader rehabilitation approaches rather than completely homogeneous treatment modalities. This residual conceptual heterogeneity may have contributed to variability in treatment effects and should be considered when interpreting comparative rankings.

Finally, the geographical distribution of the available evidence was uneven, and no eligible trials conducted in the United States were identified. The exact reasons for this finding remain unclear; however, several factors may have contributed. First, the present review applied relatively strict eligibility criteria, including the requirement that DCD be the primary diagnosis and that interventions correspond to the predefined intervention categories used in this review. Consequently, some potentially relevant studies may not have met the inclusion criteria. Second, regional differences in diagnostic practices, research priorities, and terminology related to motor coordination difficulties may also have influenced study eligibility and representation (Cairney et al., 2005b; Zwicker et al., 2012; Blank et al., 2019). Therefore, caution is warranted when generalizing the present findings to underrepresented populations and healthcare settings.

Future research should prioritize large-scale, multicenter head-to-head randomized controlled trials with standardized intervention protocols, unified outcome assessment frameworks, and extended follow-up periods. Particular attention should be given to increasing direct comparisons among active intervention approaches, improving methodological consistency, and incorporating standardized outcome measures across studies. Such efforts will strengthen the evidence base and further clarify the comparative effectiveness of different motor intervention approaches for children with DCD.

## 5 Conclusion

Based on a network meta-analysis of 20 randomized controlled trials, this study systematically compared the effects of IT, TOT, and POT on multiple motor outcomes in children with DCD. All three intervention approaches were associated with improvements in motor performance, although their relative benefits appeared to vary across functional domains. TOT demonstrated the highest probability of favorable ranking for overall motor function, whereas IT showed the highest ranking probability for balance and manual dexterity. In addition, IT and POT showed potential advantages for complex motor skills such as aiming and catching(**Supplementary Table 9**).

Subgroup analyses suggested that intervention duration may influence treatment outcomes, with interventions lasting longer than 8 weeks generally producing greater improvements. Sensitivity analyses, consistency assessments, and transitivity evaluations supported the overall robustness and validity of the network comparisons. However, notable small-study effects were identified for several outcomes, and most comparative estimates relied predominantly on indirect evidence owing to limited head-to-head trials.

Although the findings support the potential value of exercise-based interventions for improving motor function in children with DCD, confidence in comparative efficacy estimates remains limited. Evidence certainty was generally rated as low to moderate according to the CINeMA framework, and substantial clinical, methodological, and measurement heterogeneity persisted across included studies. Furthermore, discrepancies in motor assessment instruments and sparse network structures may have influenced the precision and comparability of pooled estimates. Therefore, intervention rankings and comparative advantages should be interpreted as probabilistic rather than definitive indicators of treatment superiority.

Overall, the present findings may help inform individualized intervention planning according to specific functional goals. Nevertheless, definitive conclusions regarding the superiority of any single intervention cannot yet be established. Future large-scale, multicenter head-to-head randomized controlled trials adopting standardized intervention protocols, unified outcome measures, and extended follow-up periods are required to strengthen the evidence base and further clarify the comparative effectiveness of different intervention approaches for children with DCD.

## Data Availability

The data that support the findings of this study are available from the corresponding author upon reasonable request.

## Supplementary material

Supplementary Table 1 Search strategy.

Supplementary Table 2 Classification of included interventions by therapeutic training category.

Supplementary Table 3 Results of local inconsistency test for balance function outcome (closed loop: A-C-D, corresponding to IT-POT-CG).

Supplementary Table 4 Results of local inconsistency tests for balance outcome.

Supplementary Table 5 Data sheet of sensitivity analysis by one-study removal method.

Supplementary Table 6 Leave-one-out sensitivity analysis of the pooled effect size.

Supplementary Table 7 Egger’s regression test results for different outcome indicators.

Supplementary Table 8 CINeMA assessment of confidence ratings across all network estimates.

Supplementary Table 9 Summary of recommendations for motor interventions in children with DCD based on study findings.
