# Supplementary material for: The effects of exercise intervention on children with developmental coordination disorder: a systematic review and network meta-analysis of randomized controlled trials
Source: Front Physiol. 2026 Jul 7;17:1877672. doi: 10.3389/fphys.2026.1877672 (PMC13384820; doi:10.3389/fphys.2026.1877672)
Supplement: Supplementary Table 1 — Search strategy. [file DataSheet1.docx]

# 12 Supplementary Material

S1 Table. Search Strategy

| **PubMed Search Strategy** | |
| --- | --- |
| #1 | "Motor Skills Disorders"[MeSH Terms] OR "Motor Skills Disorder"[Title/Abstract] OR "Developmental Coordination Disorder"[Title/Abstract] OR "Developmental Coordination Disorders"[Title/Abstract] |
| #2 | "Exercise"[MeSH Terms] OR "intervention"[Title/Abstract] OR "Physical Activity"[Title/Abstract] OR "exercise*"[Title/Abstract] OR "Training"[Title/Abstract] OR "sport"[Title/Abstract] OR "task-oriented training"[Title/Abstract] OR "active video games"[Title/Abstract] OR " Neuromotor Task Training "[Title/Abstract] |
| #3 | "Child"[MeSH Terms] OR "Child"[Title/Abstract] OR "children"[Title/Abstract] OR "kids"[Title/Abstract] |
| #4 | "Postural Balance"[MeSH Terms] OR "Motor proficiency"[Title/Abstract] OR "Fine Motor"[Title/Abstract] OR "Manual function"[Title/Abstract] OR "Balance"[Title/Abstract] OR "Motor coordination"[Title/Abstract] |
| #5 | "Randomized Controlled Trial"[pt] |
| #6 | #1 AND #2 AND #3 AND #4 AND #5 |

| **Cochrane Library Search Strategy** | |
| --- | --- |
| #1 | "Motor Skills Disorder" OR "Developmental Coordination Disorder" OR "Developmental Coordination Disorders" |
| #2 | Exercise OR intervention OR "Physical Activity" OR exercise* OR Training OR sport OR "task-oriented training" OR "active video games" OR "Neuromotor Task Training" |
| #3 | Child OR children OR kids |
| #4 | "Postural Balance" OR "Motor proficiency" OR "Fine Motor" OR "Manual function" OR Balance OR "Motor coordination" |
| #5 | "randomized controlled trial" OR RCT OR randomized OR randomised |
| #6 | #1 AND #2 AND #3 AND #4 AND #5 |

**1**

**2**

**3**

**4**

**5**

**6**

**7**

**8**

**9**

**10**

**11**

| **Wos Search Strategy** | |
| --- | --- |
| #1 | ("Motor Skills Disorder*" OR "Developmental Coordination Disorder*") |
| #2 | ("Exercise" OR "intervention" OR "Physical Activity" OR "exercise*" OR "Training" OR "sport" OR "task-oriented training" OR "active video games" OR "Neuromotor Task Training") |
| #3 | ("Child" OR "children" OR "kids") |
| #4 | ("Postural Balance" OR "Motor proficiency" OR "Fine Motor" OR "Manual function" OR "Balance" OR "Motor coordination") |
| #5 | ("Randomized Controlled Trial" OR RCT) |
| #6 | #1 AND #2 AND #3 AND #4 AND #5 |

| **Embase Search Strategy** | |
| --- | --- |
| #1 | 'motor skills disorder':ti,ab OR 'developmental coordination disorder':ti,ab OR 'developmental coordination disorders':ti,ab OR 'motor skills disorders'/exp OR 'motor skills disorders' |
| #2 | 'exercise':ti,ab OR 'intervention':ti,ab OR 'physical activity':ti,ab OR 'exercise*':ti,ab OR 'training':ti,ab OR 'sport':ti,ab OR 'task oriented training':ti,ab OR 'active video games':ti,ab OR 'neuromotor task training':ti,ab |
| #3 | 'child':ti,ab OR 'children':ti,ab OR 'kids':ti,ab |
| #4 | 'postural balance':ti,ab OR 'motor proficiency':ti,ab OR 'fine motor':ti,ab OR 'manual function':ti,ab OR 'balance':ti,ab OR 'motor coordination':ti,ab |
| #5 | 'randomized controlled trial' |
| #6 | #1 AND #2 AND #3 AND #4 AND #5 |

| **EBSC Search Strategy** | |
| --- | --- |
| #1 | TI "Motor Skills Disorder" OR AB "Motor Skills Disorder" OR SU "Motor Skills Disorder" OR TI "Developmental Coordination Disorder" OR AB "Developmental Coordination Disorder" OR SU "Developmental Coordination Disorder" OR TI "Developmental Coordination Disorders" OR AB "Developmental Coordination Disorders" OR SU "Developmental Coordination Disorders" OR SU "Motor Skills Disorders" |
| #2 | TI "Exercise" OR AB "Exercise" OR SU "Exercise" OR TI "intervention" OR AB "intervention" OR SU "intervention" OR TI "Physical Activity" OR AB "Physical Activity" OR SU "Physical Activity" OR TI "exercise*" OR AB "exercise*" OR SU "exercise*" OR TI "Training" OR AB "Training" OR SU "Training" OR TI "sport" OR AB "sport" OR SU "sport" OR TI "task oriented training" OR AB "task oriented training" OR SU "task oriented training" OR TI "active video games" OR AB "active video games" OR SU "active video games" OR TI "Neuromotor Task Training" OR AB "Neuromotor Task Training" OR SU "Neuromotor Task Training" |
| #3 | TI "Child" OR AB "Child" OR SU "Child" OR TI "children" OR AB "children" OR SU "children" OR TI "kids" OR AB "kids" OR SU "kids" |
| #4 | TI "Postural Balance" OR AB "Postural Balance" OR SU "Postural Balance" OR TI "Motor proficiency" OR AB "Motor proficiency" OR SU "Motor proficiency" OR TI "Fine Motor" OR AB "Fine Motor" OR SU "Fine Motor" OR TI "Manual function" OR AB "Manual function" OR SU "Manual function" OR TI "Balance" OR AB "Balance" OR SU "Balance" OR TI "Motor coordination" OR AB "Motor coordination" OR SU "Motor coordination" OR SU "Postural Balance" OR SU "Motor Skills" |
| #5 | [TI "Randomized Controlled Trial" OR AB "Randomized Controlled Trial" OR SU "Randomized Controlled Trial" OR TI "Randomised Controlled Trial" OR AB "Randomised Controlled Trial" OR SU "Randomised Controlled Trial" OR TI "RCT" OR AB "RCT" OR SU "RCT" OR TI "randomized" OR AB "randomized" OR SU "randomized" OR TI "randomised" OR AB "randomised" OR SU "randomised" OR TI "controlled trial" OR AB "controlled trial" OR SU "controlled trial"](https://research.ebsco.com/c/uqm6hk/search/advanced-results?db=a9h,ddu,bth,nlebk,e63sww,eric,hev,8gh,lxh,cmedm,nfh,bwh,trh,msn&expanders=concept&limiters=None&q=%235%20\|%20TI%20) |
| #6 | #1 AND #2 AND #3 AND #4 AND #5 |
|  |  |
| **Ovid Search Strategy** | |
| #1 | ("Motor Skills Disorder" OR "Developmental Coordination Disorder"). mp. |
| #2 | (Exercise OR intervention OR "Physical Activity" OR exercise* OR Training OR sport OR "task-oriented training" OR "active video games" OR "Neuromotor Task Training"). mp. |
| #3 | (Child OR children OR kids OR pediatric OR paediatric). mp. |
| #4 | ("Postural Balance" OR "Motor proficiency" OR "Fine Motor" OR "Manual function" OR Balance OR "Motor coordination"). mp. |
| #5 | (randomized OR randomised OR "randomized controlled trial"). mp. |
| #6 | #1 AND #2 AND #3 AND #4 AND #5 |

| **Scopus Search Strategy** | |
| --- | --- |
| #1 | "Motor Skills Disorder*" OR "Developmental Coordination Disorder*" OR dcd |
| #2 | exercise* OR intervention OR "Physical Activity" OR training OR sport OR "task-oriented training" OR "active video games" OR "Neuromotor Task Training" OR "motor imagery" OR "strength training" OR "virtual reality" OR "power training" OR "balance training" OR "task-oriented motor training" |
| #3 | child OR children OR kids OR pediatric OR paediatric |
| #4 | "Postural Balance" OR "Motor proficiency" OR "Fine Motor" OR "Manual function" OR Balance OR "Motor coordination" OR "motor performance" OR "motor skills" |
| #5 | "randomized controlled trial" OR rct OR "randomized" OR "randomised" OR "controlled trial" OR "clinical trial" |
| #6 | #1 AND #2 AND #3 AND #4 AND #5 |

| **S2 Table. Classification of included interventions by therapeutic training category** | | | | |
| --- | --- | --- | --- | --- |
| **Study** | **Intervention** | **Assigned Category** | **Primary Therapeutic Target** | **Classification Rationale** |
| Fong et al. (2016) | Functional Movement–Power Training (FMPT) | IT | Multiple motor & neuromuscular domains | This program combines standard functional task practice and lower-limb power/strength training, with both task-oriented practice and neuromuscular function improvement as core components and no single dominant therapeutic mechanism, so it is classified as Integrated Training. |
| Fong et al. (2016) | Functional Movement Training (FMT) | TOT | Functional skill acquisition & motor performance | This intervention mainly adopts repeated practice of functional balance and locomotor tasks in daily scenarios, with the core goal of directly improving motor performance through task-specific training, which fully conforms to the definition of Task-Oriented Training. |
| Ferreira et al. (2025) | Land-based functional intervention | TOT | Functional skill acquisition & motor performance | This intervention focuses on repeated practice of functional **locomotion,** balance and manipulative tasks in daily scenarios, with the core goal of directly improving children's motor performance through task-specific training, which conforms to the definition of Task-Oriented Training. |
| Ferreira et al. (2025) | Aquatic functional intervention | TOT | Functional skill acquisition & motor performance | The intervention focuses on repeated practice of functional motor tasks in an aquatic environment, with training logic identical to the land-based program. It directly targets motor skill acquisition and performance through task-specific practice, thus classified as Task-Oriented Training. |
| Wilson et al. (2016) | Motor imagery training | POT | Underlying cognitive-motor & neuromotor processes | This intervention mainly relies on action observation and mental rehearsal of movements, targeting internal motor planning and **predictive** control. It improves motor function indirectly by optimizing underlying neurocognitive processes rather than direct physical practice of functional tasks, which conforms to the definition of Process-Oriented Training. |
| Wilson et al. (2016) | Perceptual-motor training (PMT) | TOT | Functional skill acquisition & motor performance | This program consists of extensive practice of gross and fine motor tasks such as balance, ball games and manual activities, aiming to improve motor performance through repeated functional task practice, which complies with the definition of Task-Oriented Training. |
| Fong et al. (2016) | Task-specific functional balance training (FMT) | TOT | Functional balance skill acquisition & motor performance | This intervention consists of repeated practice of daily balance and locomotor tasks, aiming to **directly improve** children's balance ability and motor performance through task-specific training, which complies with the definition of Task-Oriented Training. |
| Zhang et al. (2026) | Trampoline training combined with home-based CO-OP intervention | IT | Multiple motor & cognitive domains | This combined program integrates process-oriented trampoline training and task-oriented CO-OP training, with neither component playing a dominant role and multiple complementary therapeutic mechanisms adopted to improve motor and cognitive performance, so it is classified as Integrated Training. |
| Ma et al. (2018) | Adapted Taekwondo training | IT | Multiple motor domains | This training combines functional motor task practice, balance exercise and sensory-motor function training, containing multiple therapeutic components without a single dominant mechanism, so it is classified as Integrated Training. |
| Fong et al. (2012) | Taekwondo training | IT | Balance and multi-sensory integration | This intervention combines repeated practice of functional motor tasks and stimulation of vestibular, visual and proprioceptive systems, containing multiple core training components without a single dominant therapeutic mechanism, so it is classified as Integrated Training. |
| Fong et al. (2022) | Tai chi (TC) | POT | Underlying neuromuscular function & postural control | This intervention focuses on improving basic neuromuscular control and postural stability through targeted posture and movement training. It aims to improve motor function indirectly by modifying underlying neuromuscular processes, consistent with Process-Oriented Training. |
| Fong et al. (2022) | Muscle power training (MPT) | POT | Underlying muscle strength & neuromotor function | This training targets lower-limb muscle strength and basic neuromotor control, aiming to improve motor performance indirectly by enhancing fundamental physical and neuromuscular functions, consistent with Process-Oriented Training. |
| Fong et al. (2022) | Tai chi combined with muscle power training (TC-MPT) | IT | Comprehensive motor & muscular function | This combined intervention includes multiple training components (postural training + strength training) without a single dominant focus. It targets comprehensive motor and muscular function through complementary mechanisms, thus classified as Integrated Training. |
| Fong et al. (2013) | Taekwondo training | IT | Lower limb muscle strength and static balance | This intervention combines functional motor practice, muscle training and sensory regulation training, with multiple therapeutic components integrated without a dominant mechanism, so it is classified as Integrated Training. |
| Babazadeh et al. (2025) | Life Kinetic training | IT | Balance and motor coordination | This multimodal program combines functional motor practice, perceptual and cognitive training, containing multiple core components without a dominant therapeutic mechanism, so it is classified as Integrated Training. |
| Yamanishi et al. (2025) | Ayres Sensory Integration (ASI) intervention | POT | Underlying sensory processing and neuromotor function | This intervention targets sensory integration and basic neuromotor processes, rather than direct practice of functional motor tasks. It aims to improve motor ability indirectly by optimizing underlying sensory processing mechanisms, consistent with Process-Oriented Training. |
| Wilson et al. (2002) | Motor imagery training | POT | Underlying cognitive-motor and neural processes | This intervention mainly adopts mental rehearsal and visual imagery rather than physical practice, and improves motor function by optimizing internal motor representation and neural planning, which conforms to the definition of Process-Oriented Training. |
| Wilson et al. (2002) | Traditional perceptual-motor training | TOT | Functional motor skill acquisition | This program focuses on repeated practice of gross and fine motor tasks, and directly improves motor performance through functional task training, which conforms to the definition of Task-Oriented Training. |
| Ju et al. (2018) | iBalance video game-based balance training | TOT | Functional balance skill acquisition & performance | This intervention focuses on repeated practice of functional static and dynamic balance tasks, aiming to directly improve motor performance through task-specific training, which conforms to the definition of Task-Oriented Training. |
| Hillier et al. (2010) | Aquatic physical therapy | IT | Motor skills and underlying sensorimotor function | This intervention combines functional motor task practice and stimulation of basic sensory and neuromotor processes, with multiple training components integrated without a dominant mechanism, so it is classified as Integrated Training. |
| Kordi et al. (2016) | Elastic band strength training | POT | Underlying neuromuscular function and muscle strength | This training focuses on improving muscle strength and basic neuromuscular control, rather than direct practice of functional motor tasks. It enhances motor performance indirectly by optimizing fundamental physical and neuromuscular functions, consistent with Process-Oriented Training. |
| Farhat et al. (2016) | Group-based motor skill training | TOT | Functional motor skill acquisition & performance | This intervention focuses on repeated practice of daily functional motor tasks, and directly improves motor abilities through task-specific training, which conforms to the definition of Task-Oriented Training. |
| Pless et al. (2000) | Group motor skill intervention | TOT | Functional motor skill acquisition & performance | This intervention mainly adopts repeated practice of daily functional motor tasks and games, aiming to directly improve motor performance through task-specific training, which conforms to the definition of Task-Oriented Training. |
| Coetzee & Pienaar (2013) | Visual therapy | POT | Underlying visual and oculomotor functions | This intervention mainly targets oculomotor control and visual processing abilities, and improves motor function by optimizing basic sensory and neural mechanisms rather than practicing functional motor tasks, which conforms to the definition of Process-Oriented Training. |
| Marshall et al. (2020) | Combined action observation and motor imagery training | POT | Underlying cognitive-motor and internal movement models | This intervention mainly relies on action observation and mental rehearsal instead of physical functional motor practice, and improves motor performance by optimizing internal motor representation and cognitive-motor processes, which conforms to the definition of Process-Oriented Training. |

| **S3 Table. Results of Local Inconsistency Test for Balance Function Outcome (Closed Loop: A-C-D, Corresponding to IT-POT-CG)** | | | | | | |
| --- | --- | --- | --- | --- | --- | --- |
| Closed Loop  (Loop) | Inconsistency Factor  (IF) | Standard Error  (seIF) | z-value | P-value | 95% Confidence Interval  (CI_95) | Loop-Specific Heterogeneity Variance  (τ²) |
| A-C-D | 1. 089 | 0. 979 | 1. 112 | 0. 266 | (0. 00, 3. 01) | 0. 545 |
| Note. The closed loop A-C-D corresponds to the closed evidence chain for the balance outcome in this study: Integrated Training (IT, A) - Process-Oriented Training (POT, C) - Control Group (CG, D). The inconsistency factor (IF) = 1. 089, with P = 0. 266 > 0. 05, indicating no significant inconsistency between direct and indirect evidence within this closed loop, and the consistency assumption holds. The loop-specific heterogeneity variance τ² = 0. 545 reflects the level of between-study heterogeneity within this loop. | | | | | | |

| **S4 Table. Results of local inconsistency tests for Balance outcome** | | | | | | | |
| --- | --- | --- | --- | --- | --- | --- | --- |
| side | Direct | | Indirect | | Difference | |  |
| Comparison | Coef. | Std. Err. | Coef. | Std. Err. | Coef. | Std. Err. | P > z |
| IT vs Control | 1. 9289 | 0. 5974 | — | — | — | — | — |
| TOT vs Control | 1. 7267 | 0. 6411 | — | — | — | — | — |
| POT vs Control | 1. 2885 | 0. 6343 | — | — | — | — | — |
| IT vs TOT | — | — | 0. 2022 | 0. 876 | 0. 2022 | 0. 876 | 0. 817 |
| IT vs POT | — | — | 0. 6404 | 0. 8708 | 0. 6404 | 0. 8708 | 0. 462 |
| TOT vs POT | — | — | 0. 4382 | 0. 9019 | 0. 4382 | 0. 9019 | 0. 628 |

| **S5 Table. Data Sheet of Sensitivity Analysis by One-Study Removal Method** | | | |
| --- | --- | --- | --- |
| Study omitted | Estimate | 95% Confidence Interval (Lower) | 95% Confidence Interval (Upper) |
| Maharaj S. S et al. (2016) | 1. 9555352 | 1. 5627018 | 2. 3483686 |
| Ferreira L. F et al. (2025) | 1. 9640443 | 1. 5716801 | 2. 3564086 |
| Wilson P. H et al. (2016) | 1. 9719021 | 1. 5793674 | 2. 3644369 |
| Fong S. S. M et al. (2016) | 2. 0018771 | 1. 6026776 | 2. 4010766 |
| Zhang, H et al. (2026) | 1. 9572986 | 1. 5663146 | 2. 3482828 |
| Ma, A. W. W et al. (2018) | 2. 0014265 | 1. 5958461 | 2. 4070067 |
| Fong et al. (2022) | 2. 0033851 | 1. 608222 | 2. 3985479 |
| Babazadeh et al. (2025) | 1. 8947318 | 1. 5143037 | 2. 2751598 |
| Yamanishi et al. (2025) | 1. 9324563 | 1. 5444968 | 2. 3204157 |
| Wilson et al. (2002) | 1. 9858658 | 1. 5914301 | 2. 3803017 |
| Hillier S et al. (2010) | 1. 9931614 | 1. 6025498 | 2. 3837731 |
| Farhat F et al. (2016) | 1. 8572096 | 1. 4820229 | 2. 2323961 |
| Pless M et al. (2000) | 1. 9775286 | 1. 5829849 | 2. 3720722 |
| Coetzee D et al. (2013) | 1. 9011754 | 1. 5185962 | 2. 2837546 |
| Maharaj S. S et al. (2016) | 1. 853404 | 1. 4850268 | 2. 2217813 |
| Fong S. S. M et al. (2016) | 2. 0017245 | 1. 6030756 | 2. 4003735 |
| Fong S. S. M et al. (2016) | 2. 0054126 | 1. 6082788 | 2. 4025464 |
| Fong S. S. M et al. (2016) | 1. 9862449 | 1. 5853999 | 2. 38709 |
| Zhang, H et al. (2026) | 1. 9675621 | 1. 575685 | 2. 3594391 |
| Ma, A. W. W et al. (2018) | 2. 009753 | 1. 6150697 | 2. 4044361 |
| Fong et sl. (2012) | 1. 998691 | 1. 6041453 | 2. 3932366 |
| Fong et al. (2022) | 2. 0027909 | 1. 607375 | 2. 3982067 |
| Fong et al. (2013) | 1. 9757468 | 1. 5805618 | 2. 3709319 |
| Babazadeh et al. (2025) | 1. 8916515 | 1. 5118011 | 2. 271502 |
| Yamanishi et al. (2025) | 1. 960941 | 1. 5699381 | 2. 3519437 |
| Ju et al. (2018) | 1. 92305 | 1. 5363864 | 2. 3097138 |
| Kordi H et al. (2016) | 1. 9646628 | 1. 572013 | 2. 3573124 |
| Ju et al. (2018) | 1. 9230115 | 1. 5363535 | 2. 3096695 |
| Farhat F et al. (2016) | 1. 9596766 | 1. 5678521 | 2. 351501 |
| Coetzee D et al. (2013) | 1. 9470702 | 1. 5567297 | 2. 3374107 |
| Zhang, H et al. (2026) | 1. 9456726 | 1. 5559779 | 2. 3353672 |
| Yamanishi et al. (2025) | 1. 9752836 | 1. 583424 | 2. 3671434 |
| Farhat F et al. (2016) | 1. 9507575 | 1. 5600673 | 2. 3414478 |
| Coetzee D et al. (2013) | 1. 9377465 | 1. 5489242 | 2. 3265688 |
| Maharai S. S et al. (2016) | 1. 7959183 | 1. 4393884 | 2. 1524484 |
| Zhang, H et al. (2026) | 1. 9216653 | 1. 5350991 | 2. 3082314 |
| Ma, A. W. W et al. (2018) | 2. 0079379 | 1. 6076603 | 2. 4082153 |
| Yamanishi et al. (2025) | 1. 9411194 | 1. 5521222 | 2. 3301165 |
| Farhat F et al. (2016) | 1. 9839424 | 1. 5904623 | 2. 3774223 |
| Coetzee D et al. (2013) | 1. 8984371 | 1. 5163231 | 2. 2805512 |
| Marshall B et al. (2020) | 1. 8874241 | 1. 5060463 | 2. 2688019 |
| Combined (Overall) | 1. 951303 | 1. 5664779 | 2. 3361281 |

| **S6 Table. Leave-one-out sensitivity analysis of the pooled effect size** | | |
| --- | --- | --- |
| Study omitted | Estimate | 95% Confidence Interval |
| Maharaj S. S et al. (2016) | 1. 96 | 1. 57–2. 35 |
| Ferreira L. F et al. (2025) | 1. 96 | 1. 57–2. 36 |
| Wilson P. H et al. (2016) | 1. 97 | 1. 58–2. 36 |
| Fong S. S. M et al. (2016) | 2 | 1. 60–2. 40 |
| Zhang, H et al. (2026) | 1. 96 | 1. 57–2. 35 |
| Ma, A. W. W et al. (2018) | 2 | 1. 60–2. 41 |
| Fong et al. (2022) | 2 | 1. 61–2. 40 |
| Babazadeh et al. (2025) | 1. 89 | 1. 51–2. 28 |
| Yamanishi et al. (2025) | 1. 93 | 1. 54–2. 32 |
| Wilson et al. (2002) | 1. 99 | 1. 59–2. 38 |
| Hillier S et al. (2010) | 1. 99 | 1. 60–2. 38 |
| Farhat F et al. (2016) | 1. 86 | 1. 48–2. 23 |
| Pless M et al. (2000) | 1. 98 | 1. 58–2. 37 |
| Coetzee D et al. (2013) | 1. 9 | 1. 52–2. 28 |
| Maharaj S. S et al. (2016) | 1. 85 | 1. 49–2. 22 |
| Fong S. S. M et al. (2016) | 2 | 1. 60–2. 40 |
| Fong S. S. M et al. (2016) | 2. 01 | 1. 61–2. 40 |
| Fong S. S. M et al. (2016) | 1. 99 | 1. 59–2. 39 |
| Zhang, H et al. (2026) | 1. 97 | 1. 58–2. 36 |
| Ma, A. W. W et al. (2018) | 2. 01 | 1. 62–2. 40 |
| Fong et al. (2012) | 2 | 1. 60–2. 39 |
| Fong et al. (2022) | 2 | 1. 61–2. 40 |
| Fong et al. (2013) | 1. 98 | 1. 58–2. 37 |
| Babazadeh et al. (2025) | 1. 89 | 1. 51–2. 27 |
| Yamanishi et al. (2025) | 1. 96 | 1. 57–2. 35 |
| Ju et al. (2018) | 1. 92 | 1. 54–2. 31 |
| Kordi H et al. (2016) | 1. 96 | 1. 57–2. 36 |
| Ju et al. (2018) | 1. 92 | 1. 54–2. 31 |
| Farhat F et al. (2016) | 1. 96 | 1. 57–2. 35 |
| Coetzee D et al. (2013) | 1. 95 | 1. 56–2. 34 |
| Zhang, H et al. (2026) | 1. 95 | 1. 56–2. 34 |
| Yamanishi et al. (2025) | 1. 98 | 1. 58–2. 37 |
| Farhat F et al. (2016) | 1. 95 | 1. 56–2. 34 |
| Coetzee D et al. (2013) | 1. 94 | 1. 55–2. 33 |
| Maharaj S. S et al. (2016) | 1. 8 | 1. 44–2. 15 |
| Zhang, H et al. (2026) | 1. 92 | 1. 54–2. 31 |
| Ma, A. W. W et al. (2018) | 2. 01 | 1. 61–2. 41 |
| Yamanishi et al. (2025) | 1. 94 | 1. 56–2. 33 |
| Farhat F et al. (2016) | 1. 98 | 1. 59–2. 38 |
| Coetzee D et al. (2013) | 1. 9 | 1. 52–2. 28 |
| Marshall B et al. (2020) | 1. 89 | 1. 51–2. 27 |
| Combined (full analysis) | 1. 95 | 1. 57–2. 34 |

**S1 Fig. Funnel plots for publication bias detection of each outcome**


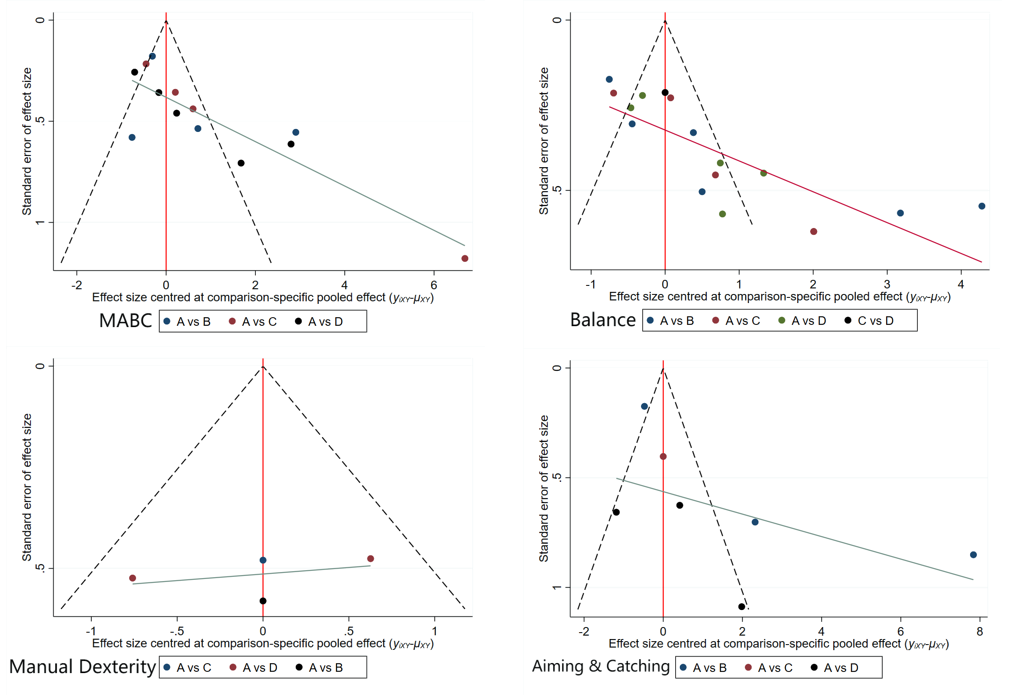


| **S7 Table. Egger’s regression test results for different outcome indicators** | | | | | | | |
| --- | --- | --- | --- | --- | --- | --- | --- |
| Outcome indicator | Number of included studies (n) | Intercept (Bias) | Standard error (Std. Err. ) | t value | P value | 95% confidence interval (95%CI) | Publication bias status |
| MABC | 14 | 4. 954 | 1. 222 | 4. 05 | <0. 001 | (2. 292,7. 616) | Significant small-study effect present |
| Balance | 16 | 6. 879 | 1. 156 | 5. 95 | <0. 001 | (4. 400,9. 358) | Significant small-study effect present |
| Aiming and Catching | 7 | 7. 16 | 1. 617 | 4. 43 | 0. 007 | (3. 002,11. 318) | Significant small-study effect present |
| Manual Dexterity | 4 | -3. 289 | 8. 809 | -0. 37 | 0. 745 | (−41. 192,34. 613) | No significant bias (insufficient power) |

| **S8 Table . CINeMA assessment of confidence ratings across all network estimates.** | | | | | | | | | |
| --- | --- | --- | --- | --- | --- | --- | --- | --- | --- |
| Outcome | Comparison | Within-study bias | Reporting bias | Indirectness | Imprecision | Heterogeneity | Incoherence | Confidence rating | Reason(s) for downgrading |
| **MABC** | CG vs IT | Some concerns | Some concerns | No concerns | No concerns | Some concerns | No concerns | Moderate | Some concerns for within-study bias, reporting bias and heterogeneity |
|  | CG vs POT | Some concerns | Some concerns | No concerns | No concerns | Major concerns | No concerns | Low | Major concerns regarding clinical and methodological heterogeneity across included studies. |
|  | CG vs TOT | Some concerns | Some concerns | No concerns | No concerns | Major concerns | No concerns | Low | Major concerns regarding clinical and methodological heterogeneity across included studies. |
|  | IT vs POT | Some concerns | Some concerns | No concerns | Major concerns | No concerns | No concerns | Low | Major concerns regarding imprecision due to wide confidence intervals and limited evidence. |
|  | IT vs TOT | Some concerns | Some concerns | No concerns | Major concerns | No concerns | No concerns | Low | Major concerns regarding imprecision due to wide confidence intervals and limited evidence. |
|  | POT vs TOT | Some concerns | Some concerns | No concerns | Major concerns | No concerns | No concerns | Low | Major concerns regarding imprecision due to wide confidence intervals and limited evidence. |
| **Balance** | CG vs IT | Some concerns | Some concerns | No concerns | No concerns | No concerns | No concerns | Moderate | Some concerns for within-study bias and reporting bias |
|  | CG vs POT | Some concerns | Some concerns | No concerns | No concerns | Major concerns | No concerns | Low | Major concerns regarding clinical and methodological heterogeneity across included studies. |
|  | CG vs TOT | Some concerns | Some concerns | No concerns | No concerns | No concerns | No concerns | Moderate | Some concerns for within-study bias and reporting bias |
|  | IT vs POT | Some concerns | Some concerns | No concerns | Major concerns | No concerns | No concerns | Low | Major concerns regarding imprecision due to wide confidence intervals and limited evidence. |
|  | IT vs TOT | Some concerns | Some concerns | No concerns | Major concerns | No concerns | No concerns | Low | Major concerns regarding imprecision due to wide confidence intervals and limited evidence. |
|  | POT vs TOT | Some concerns | Some concerns | No concerns | Major concerns | No concerns | No concerns | Low | Major concerns regarding imprecision due to wide confidence intervals and limited evidence. |
| **Manual Dexterity** | CG vs IT | Some concerns | Some concerns | No concerns | No concerns | Some concerns | No concerns | Moderate | Some concerns for within-study bias, reporting bias and heterogeneity |
|  | CG vs POT | Some concerns | Some concerns | No concerns | No concerns | Major concerns | No concerns | Low | Major concerns regarding clinical and methodological heterogeneity across included studies. |
|  | CG vs TOT | Some concerns | Some concerns | No concerns | No concerns | Major concerns | No concerns | Low | Major concerns regarding clinical and methodological heterogeneity across included studies. |
|  | IT vs POT | Some concerns | Some concerns | No concerns | Major concerns | No concerns | No concerns | Low | Major concerns regarding imprecision due to wide confidence intervals and limited evidence. |
|  | IT vs TOT | Some concerns | Some concerns | No concerns | Major concerns | No concerns | No concerns | Low | Major concerns regarding imprecision due to wide confidence intervals and limited evidence. |
|  | POT vs TOT | Some concerns | Some concerns | No concerns | Major concerns | No concerns | No concerns | Low | Major concerns regarding imprecision due to wide confidence intervals and limited evidence. |
| **Aiming and Catching** | CG vs IT | Some concerns | Some concerns | No concerns | Some concerns | Major concerns | No concerns | Low | Major concerns regarding clinical and methodological heterogeneity across included studies. |
|  | CG vs POT | Some concerns | Some concerns | No concerns | Some concerns | Major concerns | No concerns | Low | Major concerns regarding clinical and methodological heterogeneity across included studies. |
|  | CG vs TOT | Some concerns | Some concerns | No concerns | Some concerns | No concerns | No concerns | Moderate | Some concerns for within-study bias, reporting bias and imprecision |
|  | IT vs POT | Some concerns | Some concerns | No concerns | Some concerns | No concerns | No concerns | Moderate | Some concerns for within-study bias, reporting bias and imprecision |
|  | IT vs TOT | Some concerns | Some concerns | No concerns | Some concerns | No concerns | No concerns | Moderate | Some concerns for within-study bias, reporting bias and imprecision |
|  | POT vs TOT | Some concerns | Some concerns | No concerns | Some concerns | No concerns | No concerns | Moderate | Some concerns for within-study bias, reporting bias and imprecision |

| **S9 Table. Summary of recommendations for motor interventions in children with DCD based on study findings** | | | | |
| --- | --- | --- | --- | --- |
| Clinical intervention target | Preferred intervention type | Core evidence support (study data) | Common intervention characteristics (duration/frequency) | Potential applicable population |
| Improving overall motor function  (MABC) | Task-oriented training (TOT) | SUCRA=55. 3%，SMD=2. 30（95%CI：0. 42,4. 18） | ≥ 8 weeks, 2–3 sessions/week | All children with DCD, especially those requiring improvement in daily motor coordination |
| Improving balance function (SOT/mCTSIB) | Integrated training (IT) | SUCRA=78. 3%，SMD=-1. 93（95%CI：-3. 10,-0. 76） | ≥ 8 weeks, 3 sessions/week | Children with DCD with significant balance impairment and high risk of falls |
| Improving manual dexterity (Manual Dexterity) | Integrated training (IT) | SUCRA=71. 5%，SMD=-2. 11（95%CI：-4. 12,-0. 10） | ≥ 8 weeks, 2 sessions/week | Children with DCD whose fine motor deficits affect learning and daily living |
| Improving aiming and catching ability  (Aiming and Catching) | Integrated training (IT) / Process-oriented training (POT) | SUCRA=76. 6% / 75. 7%, both SMD values are significant | ≥ 10 weeks, 2 sessions/week | Children with DCD with advanced motor function impairment and difficulty in ball game participation |
| Improving overall motor function in young children (≤ 8 years) | Process-oriented training (POT)/simplified IT | Subgroup effect size SMD=1. 60, better tolerability | ≥ 8 weeks, 3 sessions/week (30 minutes per session) | Children with DCD aged ≤ 8 years (short attention span) |
| Improving balance and fine motor coordination in older children (> 8 years) | Combined IT + TOT | Subgroup effect size SMD=1. 96, higher than that in the younger age group | ≥ 10 weeks, 2–3 sessions/week | Children with DCD aged > 8 years with more complex motor demands |
